# Down the drain: exploring wastewater’s role in coastal microbiome transformations

**DOI:** 10.1186/s40168-025-02298-1

**Published:** 2025-12-24

**Authors:** Neža Orel, Eduard Fadeev, Mauro Celussi, Valentina Turk, Katja Klun, Leila Afjehi-Sadat, Gerhard J. Herndl, Tinkara Tinta

**Affiliations:** 1 Marine Biology Station Piran, National Institute of Biology, Piran, Slovenia; 2 Department of Functional and Evolutionary Ecology, University of Vienna, Vienna, Austria; 3 National Institute of Oceanography and Applied Geophysics—OGS, Trieste, Italy; 4 Mass Spectrometry Unit, Research Support Facilities, University of Vienna, Vienna, Austria; 5 NIOZ, Department of Marine Microbiology and Biogeochemistry, Royal Netherlands Institute for Sea Research, Den Burg, The Netherlands

## Abstract

**Background:**

Many coastal ecosystems worldwide are impacted by wastewater discharges, which introduce nutrients, pollutants, and allochthonous microbes that can alter microbiome composition and function. Although the severity and distribution of these impacts vary across regions, their potential consequences for key ecological processes remain a concern. The resilience and functional adaptability of native coastal microbiomes are still poorly understood. To study the immediate ecological impact of wastewater discharge on a coastal seawater microbiome, we conducted short-term microcosm experiments, exposing a coastal microbiome to two types of treated wastewater: (i) unfiltered wastewater containing nutrients, pollutants, and allochthonous microbes; and (ii) filtered wastewater containing only nutrients and pollutants.

**Results:**

By integrating multi-omics and metabolic assays, we show that wastewater-derived organic matter and nutrients (mostly ammonia and phosphate) did not alter the taxonomic composition of the coastal microbiota, but triggered reorganization of metabolic pathways in them. We observed enhanced metabolism of proteins, amino acids, lipids, and carbohydrates, particularly of the lineages Alteromonadales, Rhodobacterales, and Flavobacteriales. Glaciecola (Alteromonadales), a copiotroph with antagonistic traits, significantly contributed to these shifts. Conversely, allochthonous taxa like Legionellales and Pseudomonadales had minimal impact. Elevated phosphorus concentrations resulting from wastewater input reduced the synthesis of proteins linked to scavenging phosphorus from organic phosphorus compounds, including alkaline phosphatase activity in native Rhodobacterales and Flavobacteriales, with important ecological implications for phosphorus-depleted coastal ecosystems. Furthermore, the presence of wastewater caused a decline in relative abundance and metabolic activity of Synechococcus, potentially affecting carbon cycling. Yet, the coastal microbiome rapidly respired wastewater-derived dissolved organic carbon, resulting in bacterial growth efficiencies consistent with global coastal averages.

**Conclusions:**

Our findings highlight the capacity of coastal microbiomes to withstand wastewater discharge, with critical implications for assessment of anthropogenic perturbations in coastal ecosystems. However, wastewater-driven changes in metabolic functions and niche utilization within the autochthonous microbial community, impacting phosphorus cycling and potentially affecting carbon cycling, may have long-term consequences for ecosystem functioning.

Video Abstract

**Supplementary Information:**

The online version contains supplementary material available at 10.1186/s40168-025-02298-1.

## Background

In recent years, the inextricable link between human, animal, and environmental health has been highlighted by the One Health concept [15], emphasizing the need for holistic approaches to ensure the well-being of all three domains. Within this framework, microbes are recognized as vital for the functioning and stability of ecosystems [29], which is particularly relevant in marine environments where microorganisms represent the majority of biomass and control most biogeochemical cycles [6, 7]. Marine ecosystems provide essential ecological and economic services (e.g., nutrient cycling, pollutant degradation, food supply, and habitat provision), and emerging evidence indicates that these ecosystem services often depend on complex relationships between microorganisms and their environment [8].

However, increasing urbanization, industrial activities, and agricultural practices in coastal areas have intensified human impacts on these regions, with wastewater pollution emerging as one of the important drivers of ecological disturbances [77]. Although nearly two-third of the global wastewater produced annually receives some level of treatment [54], domestic wastewater remains a major contributor to widespread nutrient pollution in aquatic systems, the introduction of chemical pollutants and allochthonous microorganisms, including pathogens and commensal bacteria, and their antibiotic resistance genes and mobile genetic elements [40, 82]. Wastewater treatment plant effluents represent 48% of the 0.03 Tg/year total orthophosphate (P-PO_4_), 20% of the 0.11 Tg/year of total phosphorus (TP), and ~ 10% of the 1.87 Tg/year of total nitrogen (TN) annual load into the Mediterranean Sea [50]. Since P and N are limiting nutrients in many aquatic systems, disruption of their balance can have a massive impact on biodiversity and ecosystem services [26]. Despite growing concerns over wastewater-driven shifts in coastal microbiome dynamics [77], the resilience and functional adaptability of native coastal bacterioplankton remain poorly understood. Furthermore, emerging evidence shows that certain allochthonous wastewater-derived microbes can persist and remain metabolically active after release to coastal waters, especially within particle-associated or sediment microhabitats and in near-field mixing zones, where physicochemical conditions might be more favorable than in the overlaying water column [57]. While the high salinity of seawater generally poses a strong barrier to the survival and proliferation of wastewater microbes, these microhabitats may provide refugia that enable limited persistence. This creates opportunities for resource competition and other functional interaction with autochthonous microbes, raising the largely unexplored possibility that introduced taxa influence native community dynamics through niche competition or by inducing shifts in microbial physiology and behavior. However, to our knowledge, direct evidence of wastewater microbes outcompeting native seawater microbiota is lacking. Metabarcoding and advanced meta-omics approaches have emerged as powerful tools for investigating the effects of wastewater pollution on coastal microbiomes, enabling a detailed resolution of community composition, functional potential, and ecological interactions. To date, studies using 16S rRNA metabarcoding analyses have connected wastewater-polluted areas with the presence of potentially pathogenic bacteria (e.g., *Arcobacter*, *Escherichia-Shigella*, *Klebsiella*, *Salmonella*, *Vibrio*, *Clostridium*) and have revealed groups that propagate in wastewater-impacted environments (e.g., Firmicutes, Proteobacteria, Actinobacteria, and Bacteroidetes) [14, 61, 66]. In addition, few studies applying shotgun metagenomics analyses have primarily focused on gene abundances related to virulence/defense and stress response provoked by wastewater pollution, such as antibiotic resistance genes [20, 78] and the presence of mobile genetic elements in wastewater-impacted river and lake ecosystems [23, 53]. To our knowledge, no previous research has used metagenome analyses to investigate changes in the functional profiles of marine microbial communities specifically related to wastewater input. Metaproteomics advance microbial community analysis by examining expressed proteins, providing direct evidence of metabolic activity and shifts in microbial processes under changing environmental conditions [67]. This has been shown in studies addressing seasonal metabolic diversity [33], responses to algal organic matter [59], gelatinous zooplankton [28, 75], phytoplankton blooms [55], and petroleum and plastic pollution [10, 58]. So far, no comprehensive study combined a multi-omics approach to examine the influence of wastewater discharge on microbial communities in marine environments.

In this study, we conducted microcosm experiments to stimulate wastewater discharge into a coastal ecosystem. Using a combination of microbial, biogeochemical, and molecular techniques, we tested two hypotheses: (H1) that wastewater discharge alters the composition and function of coastal microbiomes; and (H2) that wastewater discharge introduces allochthonous microorganisms, including potential pathogens, which contribute to niche shifts in the autochthonous coastal microbiome.

## Results

**Fig. 1:**
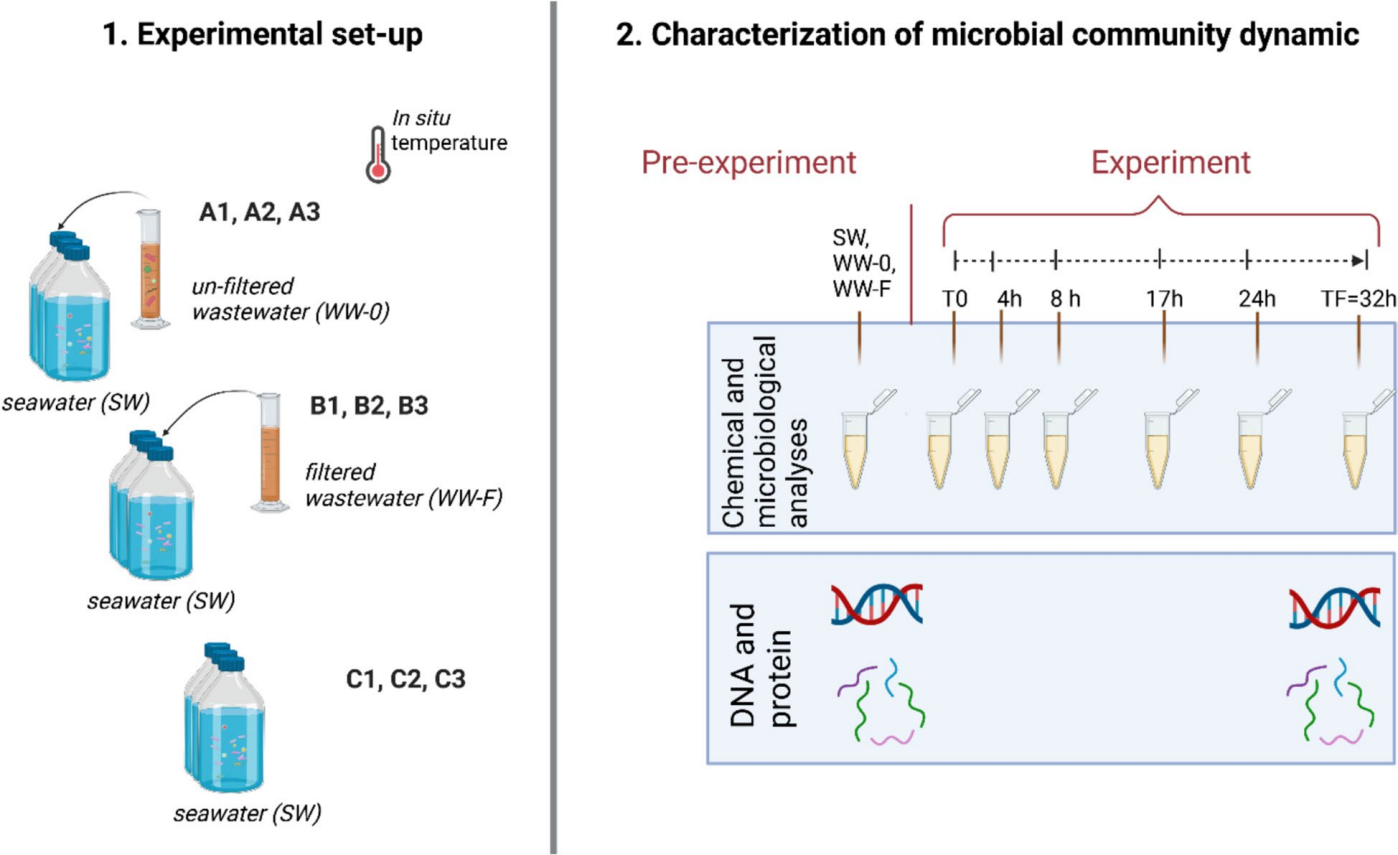
Design of the microcosm experiments. (**1.**) Each treatment was prepared in triplicate: (**A1**, **A2**, **A3**) samples with seawater (SW) and unfiltered wastewater (WW-0); (**B1**, **B2**, **B3**) samples with seawater (SW) and filtered wastewater (WW-F); and (**C1**, **C2**, **C3**) control with only seawater (SW); (**2.**) Subsampling was performed at different time points of incubation (T0, 4 h, 8 h, 17 h, 24 h, and TF = 32 h) and the experiments were terminated at the late exponential phase determined by bacterial carbon production (TF = 32 h). Analyses performed to characterize microbial community dynamics included microbiological analyses: bacterial abundance, bacterial carbon production (BCP), bacterial extracellular enzymes, and viral abundance; and chemical analyses: dissolved organic carbon (DOC), total dissolved nitrogen (TDN), and inorganic nutrients (NH_4_^+^, NO_2_^−^, NO_3_^−^, PO_4_.^3−^). Initial subsamples of the coastal seawater (SW) and unfiltered wastewater sample (WW-0) and subsamples from each experimental flask at TF were used for bacterial metagenome analyses and metaproteome analyses (except WW-0) (Created with Biorender.com)

We exposed a coastal microbiome to wastewater effluent (post-treatment, no disinfection; for details, see the “ Material and methods” section) in a short-term microcosm experiment, following the dynamics of prokaryotic community structure and function as well as fluctuations of organic and inorganic nutrients over time (Fig. 1). Treatment A was designed to assess the impact of unfiltered wastewater as a source of nutrients, chemical pollutants, and allochthonous microbes on the coastal microbiome. In treatment B, 0.2 µm prefiltered wastewater (i.e., excluding potentially introduced microbes) was used to assess the impact of wastewater as a source of nutrients and pollutants only on the coastal microbiome. Treatment C contained only seawater and served as control.

### Wastewater-introduced organic and inorganic nutrients shape dynamics of microbial abundance and metabolic activity

**Fig. 2:**
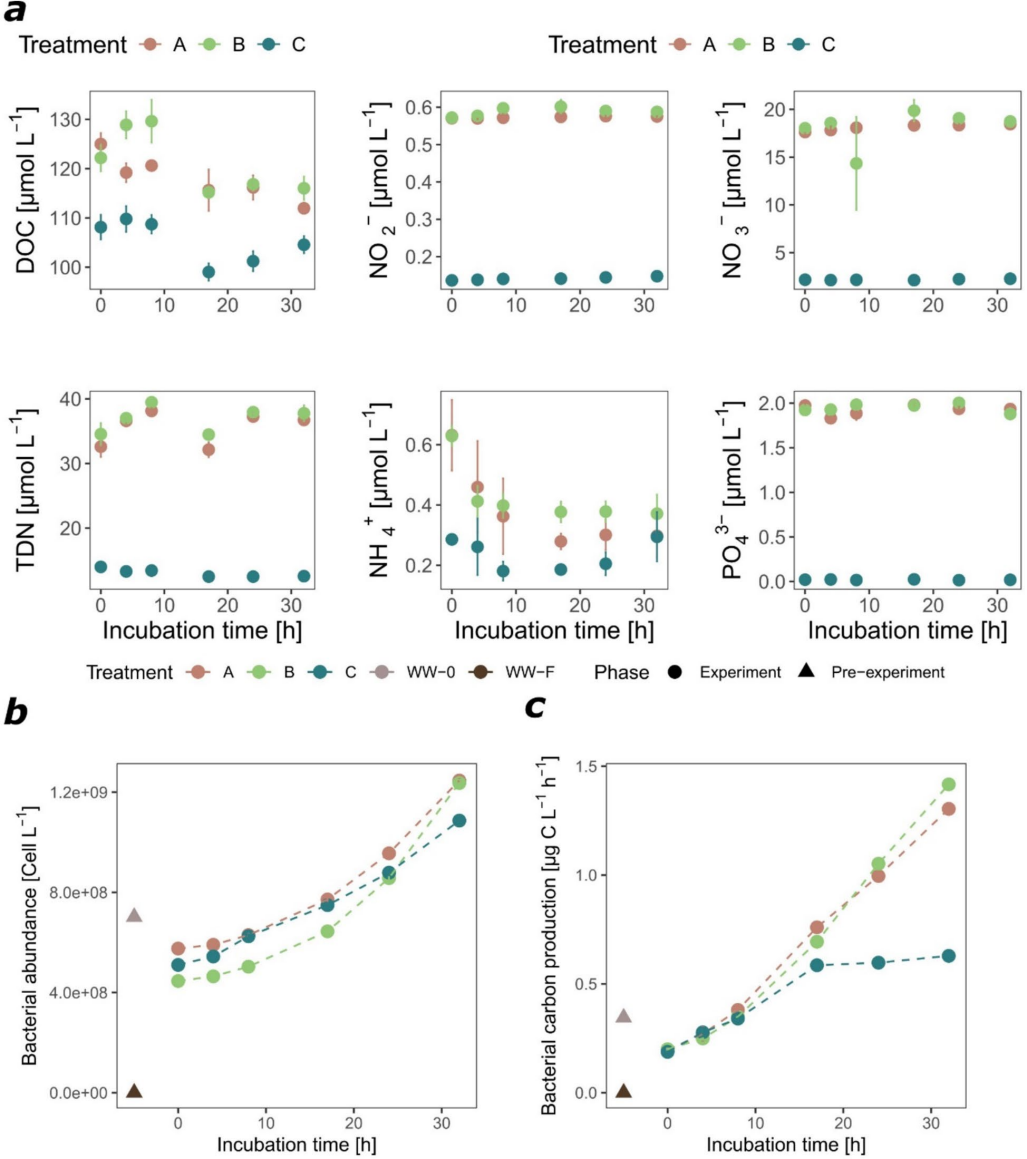
Dynamics of organic (DOC, TDN) and inorganic nutrients (NO_2_^−^, NO_3_^−^, NH_4_^+^, PO_4_.^3−^) (**a**), bacterial abundance measured by flow cytometry (**b**), and bacterial carbon production determined by incorporation of radiolabeled leucine (**c**). Measurements were taken prior to incubation for WW-0 (unfiltered wastewater) and WW-F (filtered wastewater) (triangle) and during the 32 h incubation in treatments A (seawater with unfiltered wastewater addition), B (seawater with filtered wastewater addition), and control C (seawater only) (circle). Each value represents the mean of triplicates ± standard error (SE)

Wastewater amendment introduced on average 15.5 ± 1.4 µmol L^−1^ of dissolved organic carbon (DOC), 19.6 ± 1.0 µmol L^−1^ of total dissolved nitrogen (TDN), 0.4 ± 0.01 µmol L^−1^ of NO_2_^−^, 15.7 ± 0.2 µmol L^−1^ of NO_3_^−^, 0.3 ± 0.01 µmol L^−1^ of NH_4_^+^, and 1.9 ± 0.02 µmol L^−1^ of PO_4_^3−^ (the average addition in treatments A unfiltered and B filtered at T0) (Fig. 2a). The C:N ratio of dissolved organic matter (DOM) pool in the control was 9.9. Upon wastewater addition, at 10% final concentration (for details, see “ Experimental set-up” and “ Material and methods” sections), the C:N ratio of DOM pool in the microcosms decreased by only 0.5 ± 0.4 (Supplementary Fig. 1). The dilution factor for wastewater addition was chosen to mimic real-world conditions near submarine WWTP discharges, where effluent is rapidly mixed and diluted with seawater, as demonstrated in a preliminary study from the area [49] and consistent with the order of magnitude proposed by UNEP [79]. Unfiltered wastewater amendment introduced 6.5 ± 0.1 × 10^7^ bacterial cells L^−1^ (Fig. 2b). We efficiently removed all bacterial cells by filtering wastewater (WW-F) through 0.2-µm filters and observed that filtered wastewater amendment caused on average an initial decrease by 6.4 ± 0.2 × 10^7^ bacterial cells L^−1^ in treatment B (Fig. 2b) due to dilution. Unfiltered and filtered wastewater addition also represented an inoculum of approximately 2 × 10^10^ virus-like particles L^−1^ (the average addition in treatments A and B at T0) (Supplementary Fig. 2).

During the incubation, bacterial abundance increased in all treatments reaching a slightly higher value in wastewater-treated microcosms compared to the control (Fig. 2b). At the end of the incubation (after 32 h), bacterial carbon production measured using the ^3^H-Leu incorporation method was twice as high in wastewater-treated microcosms than in the control (Fig. 2c). A decrease over time of DOC concentrations was observed in all microcosms, especially in unfiltered wastewater-treated microcosms (13.1 ± 3.0 µmol L^−1^), while in the control, the decrease was lower (3.5 ± 6.5 µmol L^−1^) (Fig. 2a). Based on the assumption that bacterial metabolism is fueled by DOC, its decrease corresponds with the heterotrophic bacterial carbon demand (BCD), which consists of bacterial production (BP) and bacterial respiration (BR). BP calculated from the increase of bacterial biomass (BB) during exponential growth was significantly different between treatments (ANOVA test with pos hoc Tukey HSD, adjusted *p*-value < 0.01), with higher values in unfiltered (A) and filtered (B) wastewater-treated microcosms than in the control (Table 1). In contrast, bacterial respiration, calculated as the difference between BCD and BP, was higher only in the unfiltered wastewater-treated microcosms (A) but not in the other treatments (Table 1). Bacterial growth efficiency (BGE) calculated as the ratio between BP (based on increase of bacterial biomass over time) and BCD was highest in filtered wastewater-treated microcosms (B) (22 ± 3%) and lowest in unfiltered wastewater-treated microcosms (A) (9 ± 1%).

**Table 1:** Bacterial community growth parameters calculated based on biological triplicates per treatment (average ± SE) (A seawater with unfiltered wastewater addition, B seawater with filtered wastewater addition, and C seawater) with *p*-values (ANOVA). The table presents values of change in growth rate (μ), bacterial biomass (BB), bacterial production (BP), bacterial respiration (BR), bacterial carbon demand (BCD), and bacterial growth efficiency (BGE)

	**A**	**B**	**C**	***p*-value**
µ (h^−1^)	0.010 ± 0.00	0.014 ± 0.00	0.010 ± 0.00	< 0.001
BB (µg C L^−1^)	13.31 ± 0.15	15.65 ± 0.14	11.22 ± 0.20	< 0.001
BP (µg C L^−1^ h^−1^)	0.42 ± 0.00	0.49 ± 0.00	0.35 ± 0.01	< 0.001
BR (µg C L^−1^ h^−1^)	4.46 ± 0.52	1.82 ± 0.26	2.40 ± 0.24	< 0.05
BCD (µg C L^−1^ h^−1^)	4.88 ± 0.52	2.31 ± 0.25	2.75 ± 0.24	< 0.05
BGE (%)	9 ± 1	22 ± 3	13 ± 1	< 0.05

We further observed a decrease in NH_4_^+^ concentrations in the wastewater-treated microcosms, while the concentrations of TDN and other inorganic nutrients did not notably change during the incubation (Fig. 2a). The C:N ratio of DOM pool decreased in wastewater-treated microcosms during the incubation (by 2.9 ± 0.6), while it remained almost constant in the control (increase by 0.8) (Supplementary Fig. 1).

**Fig. 3:**
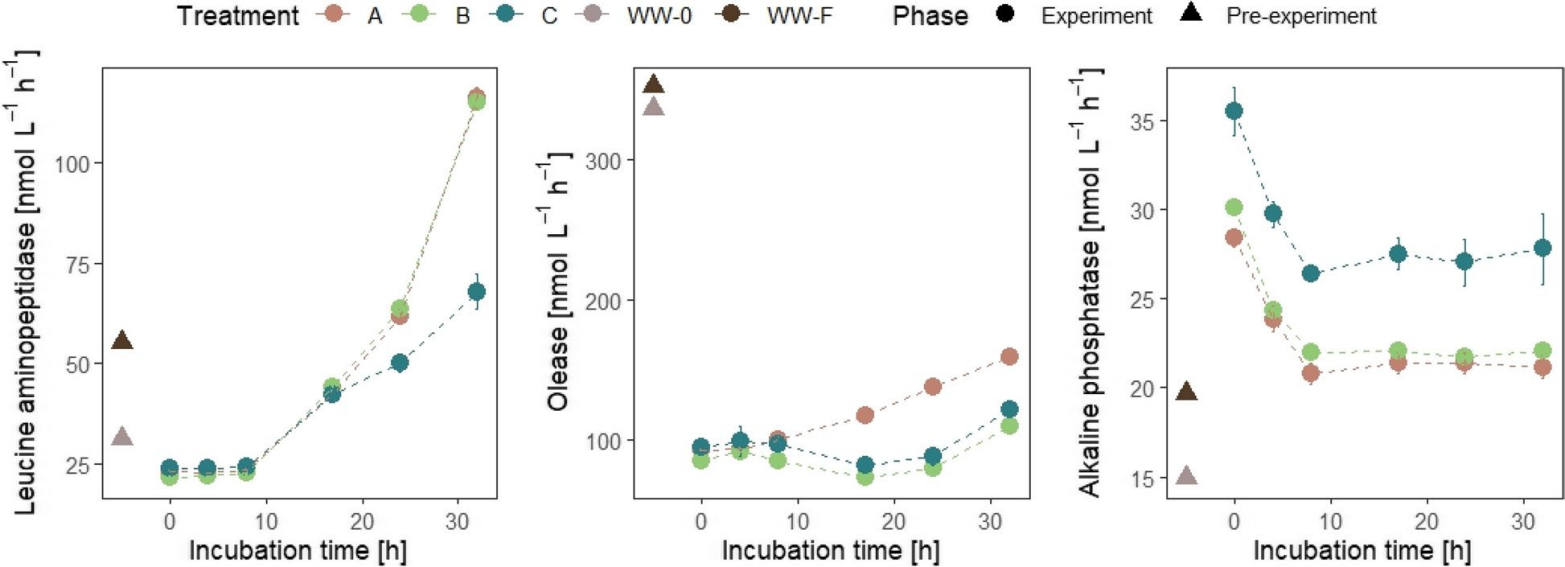
Dynamics of leucine aminopeptidase, olease, and alkaline phosphatase activity determined using fluorogenic substrate analogues prior to incubation in WW-0 (unfiltered wastewater) and WW-F (filtered wastewater) and during the 32 h incubation in treatments A (seawater with unfiltered wastewater addition), B (seawater with filtered wastewater addition), and C (seawater). Each value represents the mean of triplicates ± SE

The activity of extracellular leucine aminopeptidase was enhanced in wastewater-treated microcosms (reaching 116.3 ± 2.7 nmol L^−1^ h^−1^ and 115.2 ± 1.1 nmol L^−1^ h^−1^ in A and B, respectively) compared to the control (67.9 ± 4.4 nmol L^−1^ h^−1^) (Fig. 3). An increase was observed also for olease activity in unfiltered wastewater-treated microcosms (reaching 159 ± 2.9 nmol L^−1^ h^−1^ in A) compared to the filtered wastewater-treated microcosms and control (reaching 109.7 ± 3.2 nmol L^−1^ h^−1^ and 121 ± 5.1 nmol L^−1^ h^−1^ in B and C, respectively) (Fig. 3). However, a decrease was observed in alkaline phosphatase activity especially in wastewater-treated microcosms (from 28.4 ± 0.5 to 21.2 ± 0.7 nmol L^−1^ h^−1^ and from 30.1 ± 0.2 to 22.1 ± 0.3 nmol L^−1^ h^−1^ in A and B, respectively) compared to the control (from 35.5 ± 1.4 to 27.8 ± 2.0 nmol L^−1^ h^−1^) (Fig. 3). β-glucosidase and chitinase remained nearly constant (Supplementary Fig. 3).

### Wastewater does not markedly restructure coastal microbiomes

We analyzed the bacterial metagenomes and metaproteomes in each microcosm at the late exponential bacterial growth phase (32 h) as well as in the coastal seawater (SW) collected prior to the start of the experiment (Fig. 1). We were unable to obtain sufficient biomass from unfiltered wastewater sample (WW-0) for metaproteomic analysis: attempts to concentrate material onto 0.2-µm filters consistently led to filter clogging, preventing efficient protein recovery. Due to this technical challenge, we were only able to extract DNA from the unfiltered wastewater sample (WW-0). Technical details of metagenomic and metaproteomic datasets and analyses are presented in the supplementary information (Supplementary Text S1, Supplementary Tables S1–S2, Supplementary Figures S4–S6). Our short-term microcosm design was not intended to distinguish between stable and variable components of the microbiome. Such patterns are more appropriately resolved in long-term or repeated in situ studies that capture temporal dynamics and environmental variability. Here, our focus was to assess short-term responses of coastal microbial communities to wastewater amendment.

**Fig. 4:**
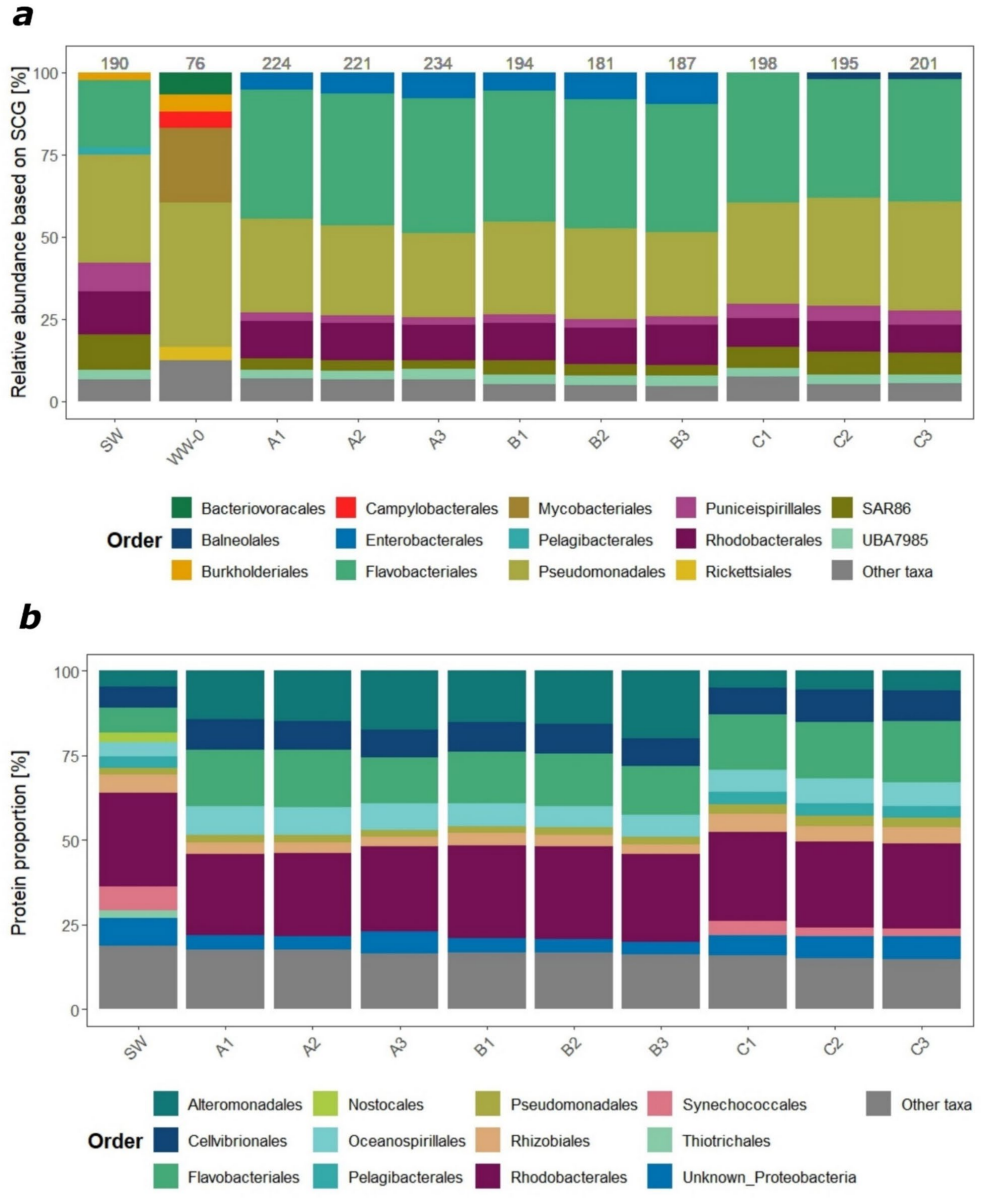
Microbial community composition based on (**a**) coverage of SCG gene (Ribosomal S2) in the metagenomic dataset and (**b**) protein abundance (NSAF-transformed values) in the metaproteomic dataset assigned to bacterial orders. Samples include the coastal microbiome (SW) and wastewater microbiome (WW-0) collected prior to the experiments and microbiomes from treatments after 32 h of incubation: A1–A3 (triplicates of seawater with unfiltered wastewater addition), B1–B3 (triplicates of seawater with filtered wastewater addition), and control C1–C3 (triplicates of seawater only). Bacterial orders with a relative abundance < 2% or unknown taxonomy on the order level are joined as “Other taxa.” Numbers at the top (**a**) represent the count of ribosomal S2 genes in each sample

Based on ribosomal S2 single-copy gene (SCG) taxonomic profiling of metagenomics data (Fig. 4a) (for rationale, see “ Material and methods” section), the coastal microbiome (SW) was dominated by the orders Pseudomonadales (32% of SCGs), followed by Flavobacteriales (20% of SCGs) and Rhodobacterales (13% of SCGs). These orders remained prevalent in all treatments (A, B, C) during the late exponential phase (Fig. 4a). Enterobacteriales were considerably more abundant in the wastewater treatments (approximately 7% and 8% of SCGs in treatments A and B, respectively) compared to the control (C, < 1% of SCGs). Pseudomonadales also dominated the wastewater (WW-0) microbiome (44% of SCGs), followed by groups not detected in seawater, such as Mycobacteriales (23% of SCGs), Bacteriovoracales (7% of SCGs), Campylobacterales (5% of SCGs), and Rickettsiales (4% of SCGs) (Fig. 4a). Mycobacteriales and Campylobacterales were detected only in the unfiltered wastewater-treated microcosms (A), each comprising up to 1% of SCGs (Fig. 4a).

The coastal metaproteome (SW) was dominated by Rhodobacterales (28%), followed by Flavobacteriales (7%) and Synechococcales (7%), based on protein abundance calculated from NSAF-transformed values (Fig. 4b). Complementary to the metagenome results, proteins of Rhodobacteriales and Flavobacteriales origin represented a substantial fraction (25 ± 1% and 16 ± 1%, respectively) of the metaproteomes extracted at the late exponential bacterial growth phase from all microcosms (A, B, C) (Fig. 4b). The proportion of proteins taxonomically assigned to Alteromonadales (taxonomically assigned as Enterobacteriales in metagenomes, reflecting known discrepancies between databases; illustrating the complexity of MAG taxonomic affiliations, see further explanation in the “ Material and methods” section) was higher in the wastewater-treated microcosms (A and B on average 16 ± 1%) than in the control (C) (6%). Campylobacteriales represent only a small proportion of proteins detected in the unfiltered wastewater treatment (< 0.5%) while proteins from Mycobacteriales were not detected (Fig. 4b).

**Fig. 5:**
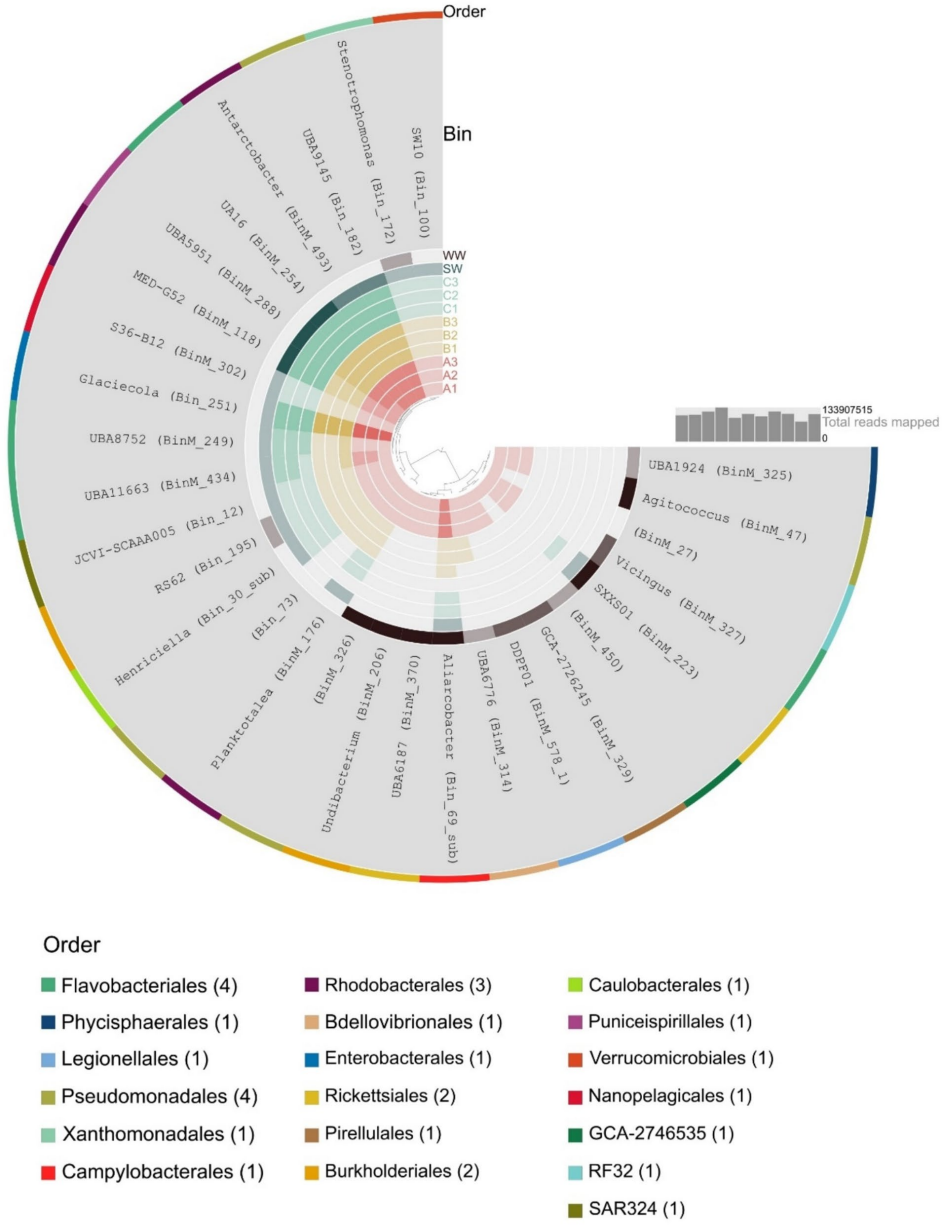
Visualization of MAGs detected in different samples. The color code in inner circles represents treatment (A-red, B-yellow, C-green, SW-blue, WW-0-brown) and intensity represents the read coverage of specific MAG in samples (thresholds: 0, 0–5, 5–10, 10–100, > 100)

From the co-assembled metagenome, we were able to reconstruct a total of 29 metagenome assembled genomes (MAGs) larger than 1.3 Mbp in total length, > 80% completeness, and < 12% redundancy (Supplementary Table S3). Taxonomic profiling of the MAGs revealed their affiliation with Flavobacteriales, Pseudomonadales, and Rhodobacterales (4, 4, and 3 MAGs, respectively) as well as with other taxonomic lineages (Fig. 5). A total of 9 MAGs, affiliated with Rickettsiales, Flavobacteriales, Pseudomonadales, Legionellales, Burkholderiales, Phycisphaerales, Pirellulales, and JAEUKQ01 (order within Alphaproteobacteria) (Fig. 5), were detected only in the initial wastewater sample (WW-0) and unfiltered wastewater-treated microcosms (A).

The MAGs with higher read coverage in the wastewater-treated microcosms, compared to the control, were affiliated with Enterobacteriales, Bdellovibrionales (A and B treatment), and Campylobacteriales (only A treatment) (Fig. 5). A particularly high read coverage was observed for Bin_251, taxonomically affiliated with *Glaciecola*, in wastewater treatments (reads coverage 331 and 722 in A and B, respectively) compared to the control (coverage 15–21) (Fig. 5, Supplementary Fig. 7). To further explore this MAG, we constructed a pangenome with its taxonomically related reference genomes (based on GTDB-Tk) (Supplementary Table S4). The closest reference genome to the *Glaciecola* MAG was *Glaciecola sp000155775* (GCF_000155775.1) (ANI 80.63%), both sharing 685 genes with all other reference genomes (core genes) (729 genes in soft core gene cluster, shared between the majority of genomes) and 56 genes not present in other genomes (unique) (Supplementary Fig. 8). Most proteins in the metaproteomic dataset were assigned to the *Glaciecola* Bin_251 (Supplementary Fig. 9) (8 ± 2% of all proteins associated with any MAGs in treatments with wastewater (A and B) and < 1% in the control and seawater inoculum).

### Wastewater alters the function of key microbial taxa mediating biogeochemical processes in coastal ecosystems

**Fig. 6:**
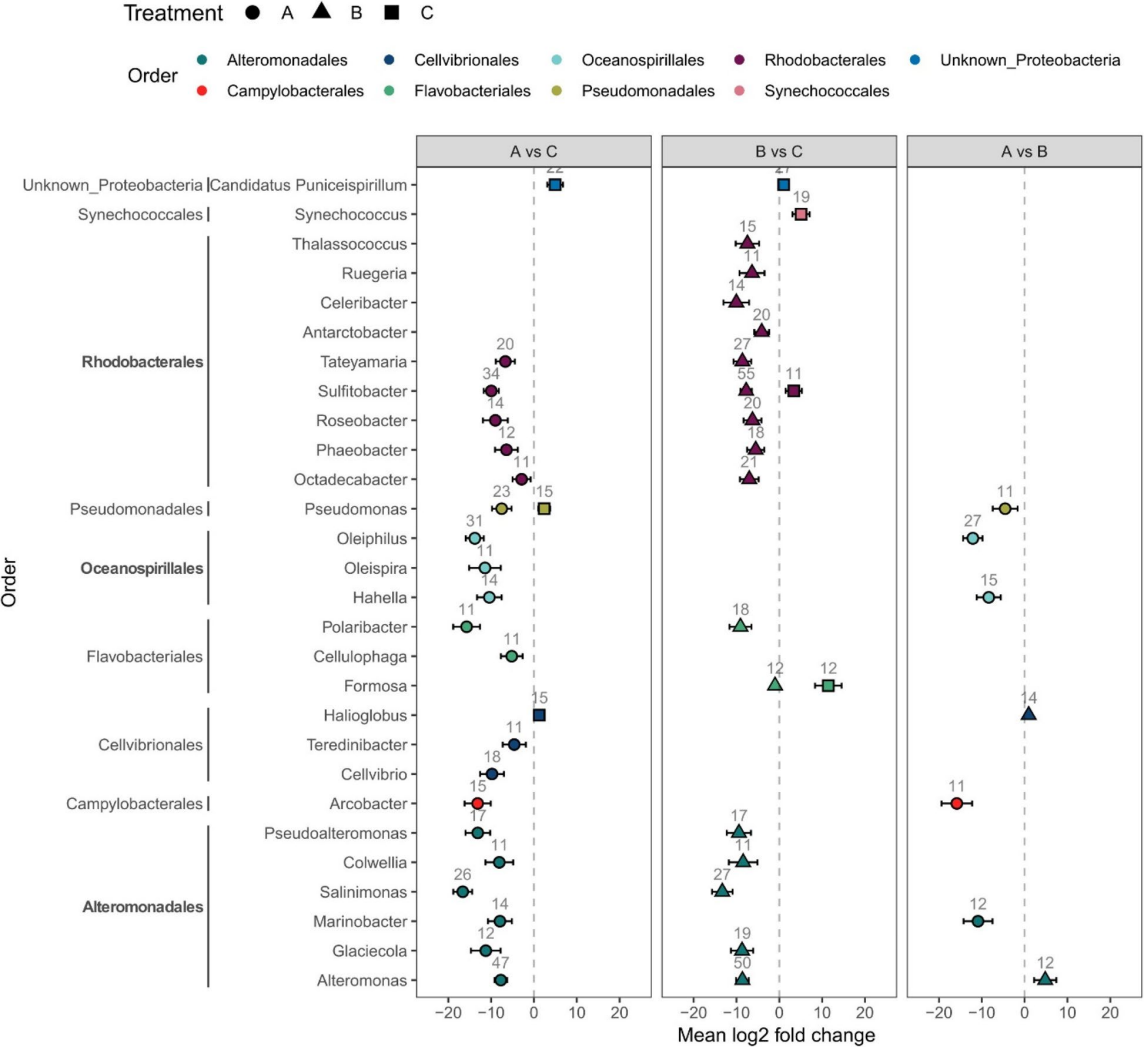
Differential abundance of proteins according to taxonomic annotation. Comparisons include unfiltered wastewater-treated microcosm versus control (A vs C), filtered wastewater-treated microcosm versus control (B vs C), and unfiltered versus filtered wastewater-treated microcosm (A vs B). Only taxonomic groups with more than 10 proteins and an absolute mean log2 fold change greater than 3 are shown

**Fig. 7:**
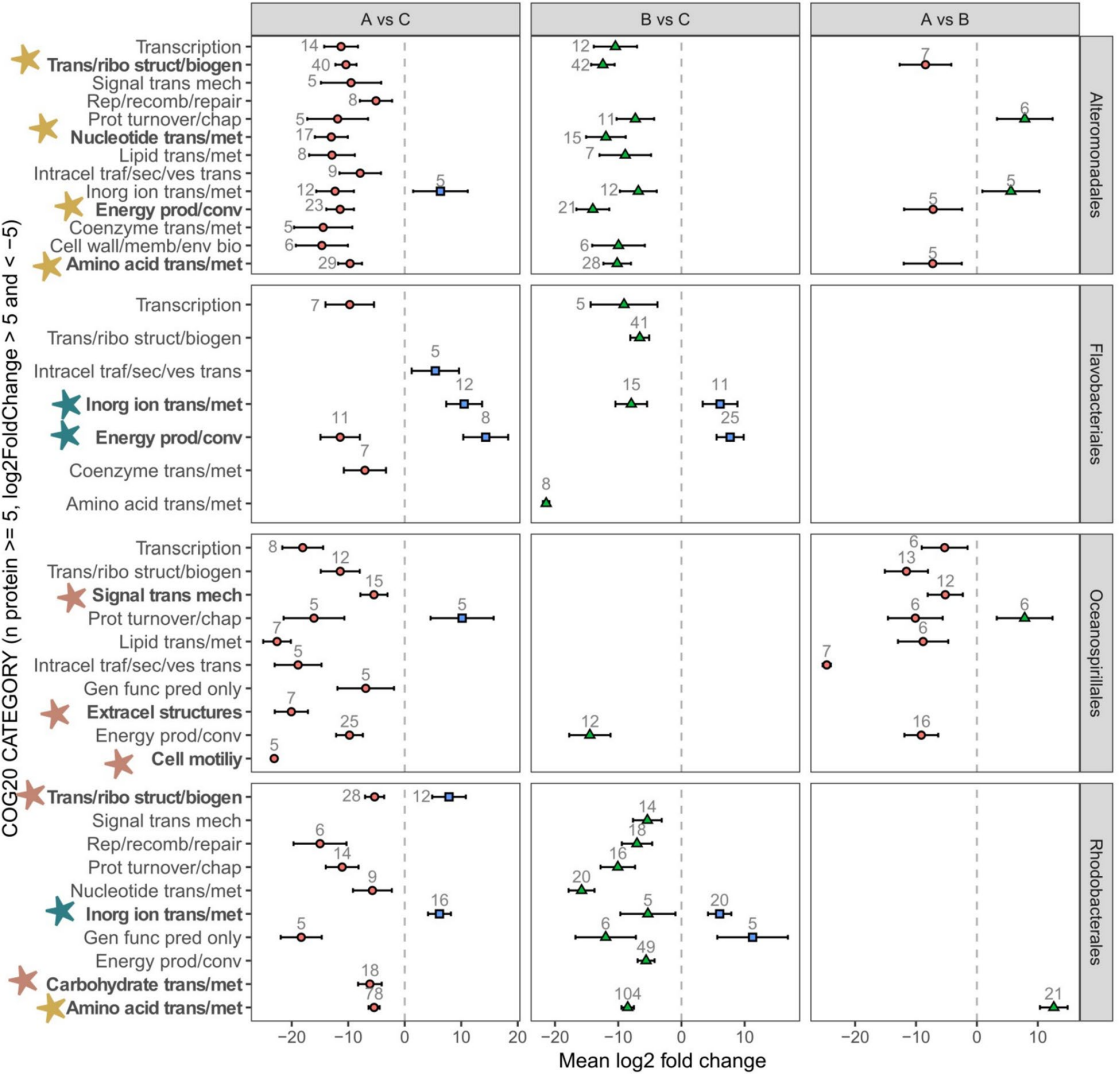
Differential protein abundance across COG categories in dominant taxonomic groups of wastewater-treated microcosms. Changes in protein abundance were analyzed in unfiltered wastewater vs control (A vs C), filtered wastewater-treated microcosm vs control (B vs C), and unfiltered vs filtered wastewater-treated microcosm (A vs B). Only COG (clusters of orthologous groups) categories with > 5 proteins and an absolute mean log_2_ fold change > 5 are displayed. Selected COG categories are highlighted with stars: red asterisks indicate enrichment in unfiltered wastewater-treated microcosms compared to control (A vs C), yellow asterisks indicate enrichment in both wastewater-treated microcosms compared to control (A vs C and B vs C), and blue asterisks indicate enrichment in the control compared to both wastewater-treated microcosms (C vs A and C vs B). COG category abbreviations: Amino acid trans/met (amino acid transport and metabolism; E); Cell wall/memb/env bio (cell wall/membrane/envelope biogenesis; M); Coenzyme trans/met (coenzyme transport and metabolism; H); Energy prod/conv (energy production and conversion; C); Inorg ion trans/met (inorganic ion transport and metabolism; P); Intracel traf/sec/ves trans (intracellular trafficking, secretion, and vesicular transport; U); Lipid trans/met (lipid transport and metabolism; I); Nucleotide trans/met (nucleotide transport and metabolism; F); Prot turnover/chap (posttranslational modification, protein turnover, chaperones; O); Rep/recomb/repair (replication, recombination, and repair; L); Signal trans mech (signal transduction mechanisms; T); Transcription, Trans/ribo struct/biogen (translation, ribosomal structure, and biogenesis; J); Cell motility (N); Extracel structures (extracellular structures; W); Gen func pred only (general function prediction only; R); Carbohydrate trans/met (carbohydrate transport and metabolism; G)

We identified 884 and 817 bacterial proteins that were enriched in response to wastewater amendment (A vs C and B vs C, respectively) (adjusted *p* value < 0.1, Supplementary Table S5). However, we cannot fully exclude the possibility that some of these proteins also reflect the inherent proteomic profile of the effluent wastewater itself. Among those enriched in both wastewater-treated microcosms, we identified mostly proteins taxonomically affiliated to Alteromonadales (*Glaciecola*, *Pseudoalteromonas*, *Colwellia*, *Salinimonas*, *Alteromonas*), Flavobacteriales (*Polaribacter*), and Rhodobacterales (*Octadecabacter*, *Phaeobacter*, *Roseobacter*, *Sulfitobacter*, *Tateyamaria*) (Fig. 6). Enriched proteins of Alteromonadales and Rhodobacteriales were largely associated with amino acid metabolism (Fig. 7), especially with glutamine and glutamate synthase (GltD, GltB1, GltB1, GltB1) and ABC-type amino-acid transport systems (e.g., LivK, LivF) (Supplementary File 2). Other enriched proteins of Alteromonadales in the wastewater-treated microcosms were associated with lipid metabolism (Fig. 7) (especially fatty acid β-oxidation – FadB, CaiA, CaiD) (Supplementary File 2).

Some taxa, such as Campylobacteriales (*Arcobacter*), Cellvibrionales (*Teredinibacter*, *Cellvibrio*), Oceanospirillales (*Oleiphilus*, *Oleispira*, *Hahella*), and some representatives of Alteromonadales (*Marinobacter*) and Pseudomonadales (*Pseudomonas*), contributed to the significant enrichment of proteins in the unfiltered wastewater-treated microcosms as compared to the control (A vs C, Fig. 6). Proteins of Oceanospirillales origin were related to cell motility and extracellular structures (e.g., pilus proteins PilT), associated with signal transduction mechanisms (e.g., chemotaxis proteins—CheY, CheW, CheA, Tar), and related to lipid transport and metabolism (e.g., FadB, PaaJ, CaiA, CaiD, AccB) (Fig. 7, Supplementary File 2). In unfiltered wastewater-treated microcosms, we also recorded an enrichment of carbohydrate transport and metabolism proteins of Rhodobacteriales origin (e.g., transketolase TktA, transport system proteins DctP, UgpB, RbsB) (Fig. 7, Supplementary File 2).

Proteins of Campylobacteriales (*Arcobacter*), Alteromonadales (*Marinobacter*), Oceanospirillales (*Oleiphilus*, *Hahella*), and Pseudomonadales (*Pseudomonas*) were also significantly enriched in unfiltered vs filtered wastewater-treated microcosms (A vs B, Fig. 6) and were mainly related to intracellular trafficking, secretion, and vesicular transport (e.g., Type II secretory pathway component HofQ, PulD, PulL, pilus assembly protein TadD), signal transduction mechanisms (chemotaxis protein CheW, CheY, CheA), lipid transport and metabolism (CaiA, FadB, CaiD), and cell motility (pilus assembly protein PilA) (Fig. 7, Supplementary File 2).

In contrast, no proteins were uniquely enriched in the filtered wastewater-treated microcosms (B vs C) that were not also enriched in the unfiltered wastewater-treated microcosms (A vs C).

In the control (A vs C and B vs C), we recorded enrichment in proteins related to inorganic ion transport of Flavobacteriales and Rhodobacterales origin (Fig. 7). Proteins of Rhodobacterales origin were enriched in phosphate transporters (phosphate uptake PhoU, phosphate transporters PstS, PhnD, alkaline phosphatase PhoA) (Supplementary File 2). In addition, in the control, we recorded an enrichment of proteins of Synechococcales (mainly *Synechococcus*) origin (Fig. 6) that were mainly related to energy production and conversion (photosystems I and II) (Supplementary File 2).

### Wastewater provokes niche shifts among key members of the coastal microbiome

**Fig. 8:**
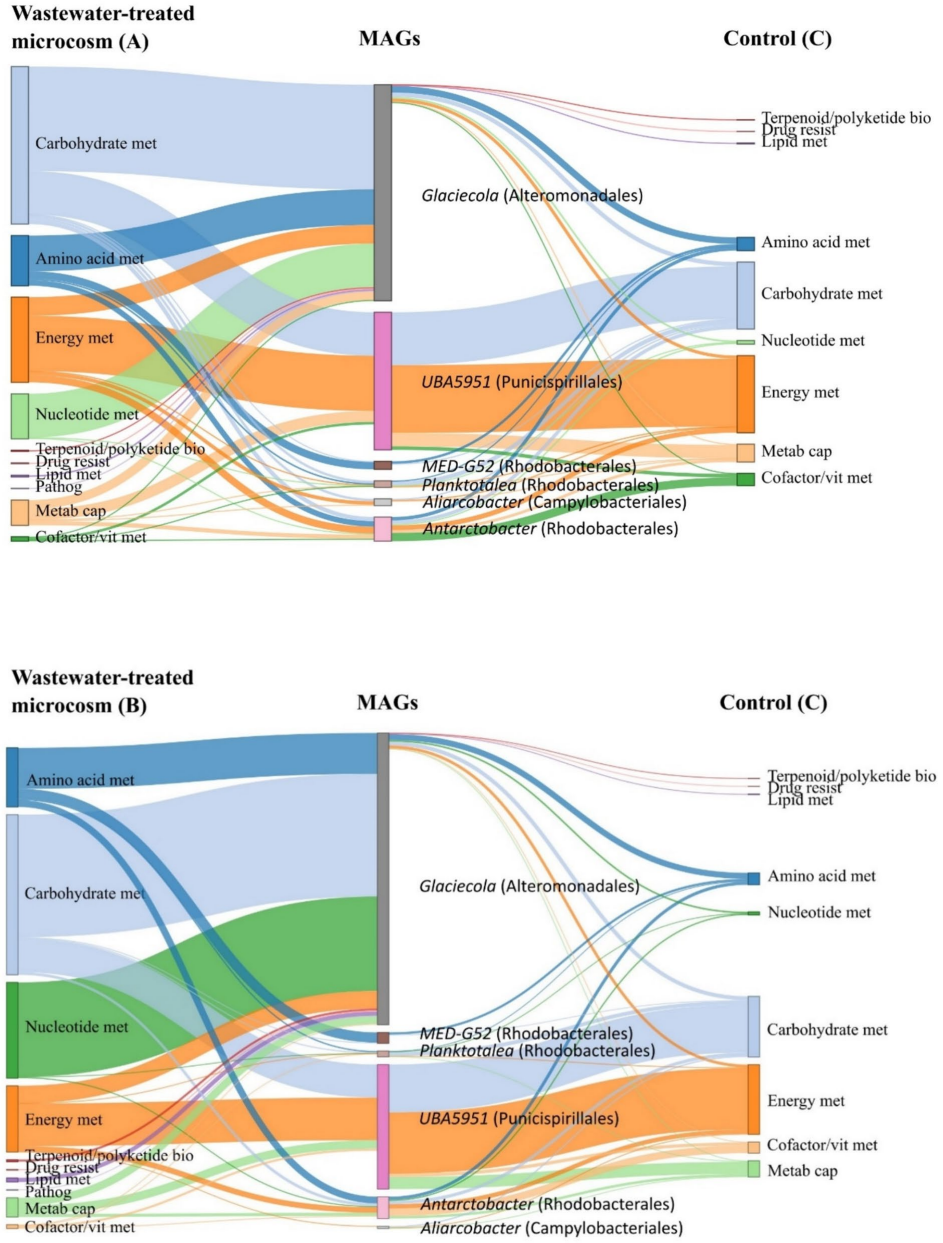
Sankey diagram illustrating metabolic functions across MAGs under different treatments. The diagram depicts the association of proteins with metagenome-assembled genomes (MAGs) under different treatments. Nodes on the left represent proteins from wastewater-treated microcosms (**A** or **B**), nodes in the middle correspond to MAGs, and nodes on the right represent proteins in the control (**C**). Proteins are categorized by Kyoto Encyclopedia of Genes and Genomes (KEGG) functional pathways, with color-coded flows indicating KEGG categories. Each link represents a protein assigned to a KEGG category within a specific MAG, with link thickness reflecting protein abundance (NSAF-transformed values). Only protein groups with an abundance > 0.00001 are displayed. KEGG category abbreviations: Amino acid met (amino acid metabolism), Carbohydrate met (carbohydrate metabolism), Energy met (energy metabolism), Nucleotide met (nucleotide metabolism), Lipid met (lipid metabolism), Cofactor/vit met (metabolism of cofactors and vitamins), Terpenoid/polyketide bio (biosynthesis of terpenoids and polyketides), Drug resist (drug resistance), Pathog (pathogenicity), Metab cap (metabolic capacity)

In the metaproteomic dataset, we identified proteins linked to 24 MAGs. The number of proteins connected to MAGs was 1015 in unfiltered wastewater-treated microcosms (A), 1034 in filtered wastewater-treated microcosms (B), and 749 in the control (C). However, in different treatments, specific MAGs exhibited different protein profiles (Fig. 8). Proteins of *Glaciecola* (Bin_251, Alteromonadales) showed a higher proportional abundance of proteins in wastewater treated microcosms (A and B) (0.31 and 0.23, respectively) compared to the control (0.09) (C), with the majority of proteins related to amino acid metabolism, carbohydrate metabolism, and nucleotide metabolism according to KEGG annotation. In addition to these core metabolic functions, proteins of *Glaciecola* (Bin_251) related to drug resistance, lipid metabolism, and pathogenicity were more expressed in wastewater-treated microcosms. In contrast, proteins of Puniceispirillales representatives (BinM_288, UBA5951) exhibited a comparable abundance (~ 0.02) and were involved in similar processes (carbohydrate metabolism, energy metabolism) across all enclosures, suggesting that this taxon may maintain a relatively stable metabolic profile under the experimental conditions tested. While this could indicate metabolic stability, we caution that the absence of detectable changes may also reflect limitations in experimental duration, sensitivity of proteomic detection, or unmeasured regulatory processes. Likewise, a representative of Rhodobacterales (BinM_493, *Antarctobacter*) was maintaining similar metabolic processes in all enclosures (exhibiting a comparable abundance of proteins 0.09, 0.14, 0.14; in A, B, and C respectively). In the control (C), however, an increased representation of proteins was detected related to cofactor and vitamin metabolism, potentially linked to enhanced alkaline phosphatase activity.

## Discussion

We simulated a scenario of wastewater treatment plant (WWTP) discharge into a coastal ecosystem in short-term microcosm experiments. The WWTP discharge amendment resulted in a significant increase in dissolved organic carbon (DOC), nitrogen, and inorganic nutrients concentration in our microcosms (Fig. 2). This level of nutrient amendment is especially relevant in phosphorus (P)- and nitrogen (N)-limited environments such as our study ecosystem, the northern Adriatic Sea, which triggered an elevated bacterial activity. We observed that bacterial carbon production reached two times higher values in the wastewater–treated microcosms compared to the control after a 32 h incubation, coupled with a decline in DOC and NH₄⁺ concentrations. This indicates that the microbiomes rapidly responded to the newly introduced nutrients. In response to the increased DOC concentrations from the wastewater input, bacterial respiration rates exceeded biomass production. This resulted in a low bacterial growth efficiency (BGE of 9–22%), comparable to that of bacterioplankton in other coastal ecosystems (27 ± 18%, [34]). These results suggest that the coastal microbiome may buffer against wastewater-derived organic matter (i.e., DOC) that is rapidly respired and released as dissolved CO_2_, potentially due to the adaptation to this type of allochthonous substrates in an anthropogenically impacted coastal ecosystem. Alternatively, the fact that the wastewater-derived DOC primarily sustained only basal metabolism in the microcosms indicates its low-quality as substrate for bacterial growth and/or may be due to the final wastewater concentration of 10% (which aimed at simulating rapid dilution upon discharge into the water column, see the “ Material and methods” section) that resulted in minimal changes to the overall DOM stoichiometry in microcosms. In this study, we did not analyze specific chemical compounds, potentially introduced by wastewater, such as organic pollutants and antibiotics. These compounds may have significant implications for functioning of coastal microbiome [78] and should be a focus of future research.

The observed preferential uptake of NH_4_⁺ over NO_3_⁻, consistent with patterns in other coastal and riverine systems [5, 24], indicates that NH_4_⁺ is the preferred nitrogen source for coastal microbiomes. High levels of organic nitrogen, inorganic phosphate, and nitrate and nitrite (NO_2_^−^ + NO_3_^−^) persisted in the wastewater-treated microcosms throughout the experiment. This suggests a continuous leaching of dissolved organic and inorganic nitrogen from the particulate fraction due to microbial transformation and re-mineralization processes. At the same time, persistently high inorganic phosphorus levels coupled with low alkaline phosphatase activity indicates a minimal contribution of phosphorus requirements from organophosphorus compounds. Thus, suggesting that wastewater-derived phosphorus might sustain bacterial communities for some time, which has important implications for phosphorus-limited ecosystems.

**Fig. 9:**
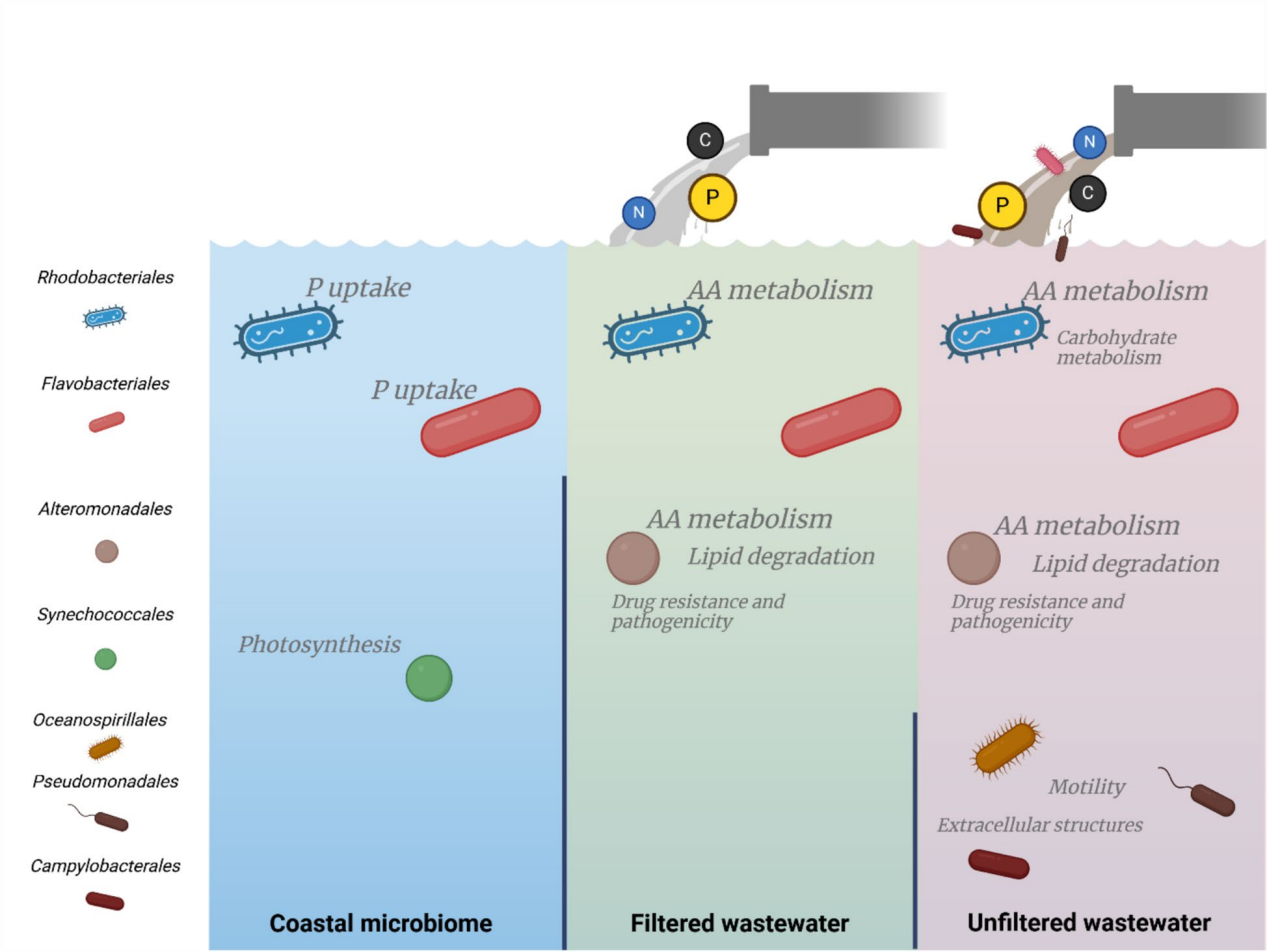
Schematic representation of the results. The left panel illustrates coastal seawater and the associated coastal microbiome (control C), the middle panel represents seawater and the associated coastal microbiome exposed to filtered wastewater as a source of nutrients (treatment B), and the right panel depicts seawater and the associated coastal microbiome exposed to wastewater as a source of both nutrients and allochthonous bacteria (treatment A). Highlighted are the enriched metabolic processes mediated by key microbial taxa in each treatment

Our multi-omics approach revealed that wastewater addition did not markedly alter the overall composition of the enclosed coastal microbiomes with most recovered proteins across all treatments originating from the same taxa, Rhodobacteriales, Flavobacteriales, and Alteromonadales. However, the organic compounds and inorganic nutrients introduced via the wastewater altered metabolic processes operated by these taxa (Figs. 4, 5, and 9) resulting in enhanced metabolism of proteins, amino acids, lipids, and carbohydrates as indicated by our proteomic analysis and measurements of extracellular enzymatic activities (Figs. 3, and 7). Leucine aminopeptidase activity was elevated in all wastewater treatments, with Alteromonas being key degraders of proteinaceous compounds introduced with wastewater, as suggested by enrichment analysis of protein profiles (Figs. 3, and 7). Alteromonadales are copiotrophic bacteria with a high growth potential when organic nutrient concentrations are elevated—e.g., they are frequently associated with the decay phases of phytoplankton [43, 74] and gelatinous zooplankton [28, 75] blooms, where they play a key role in the degradation of complex organic compounds. However, in the sampled coastal ecosystem, they are not a typical representative of the core microbiome [17, 61]. Nonetheless, Alteromonadales have been previously linked to degradation and utilization of polycyclic aromatic hydrocarbons and other types of anthropogenic dissolved organic carbon [18, 36, 68], which may explain their elevated activity. Alteromonadales, together with Rhodobacteriales, were involved in amino acid metabolism in the wastewater-treated microcosms, as indicated by the overexpression of enzymes involved in glutamine and glutamate synthesis and ABC-type amino acid transport systems (Figs. 6, and 7). Our analysis suggests that the drawdown of wastewater-derived NH_4_^+^ could be largely driven by Alteromonadales and Rhodobacterales as indicated by the enrichment of glutamine synthesis in their protein profiles (Figs. 2, and 7), a primary pathway for ammonium assimilation in microbial communities [32]. Additionally, microbes rapidly responded to nutrient influx by increased synthesis of ABC-type amino acid transport systems. Linking the proteomic data with reconstructed metagenome-assembled genomes (MAGs) revealed that most of the proteins in wastewater-treated microcosms were affiliated with a single MAG of Glaciecola (Alteromonadales). These proteins were primarily associated with amino acids, carbohydrates, and lipids metabolism.

Our metaproteomic data revealed a marked enrichment of proteins related to lipid metabolism in both wastewater-treated microcosms, in particular, but not limited to proteins involved in fatty acid β-oxidation. Alteromonadales were the key contributors to lipid metabolism in wastewater treatments, while Oceanospirillales played a significant role only in the unfiltered wastewater–treated microcosms. Notably, olease activity, a proxy for lipid degradation processes, was elevated only in the unfiltered wastewater treatment. This discrepancy may be due to a difference in the resolution of metabolic measurements and multi-omics methods. While enzymatic activity measurements provide insights into the ability of microbes to degrade a hypothetical substrate, serving as a proxy for natural substrates, omics approaches categorize “lipid metabolism” more broadly, encompassing a wider array of proteins involved in lipid-related metabolic processes. Alternatively, the observed difference could reflect lipid degradation of particulate matter introduced with unfiltered wastewater, or suggesting an important role of allochthonous microbes from unfiltered wastewater in lipid processing. However, taxa such as Legionellales, Flavobacteriales, Pseudomonadales, and Burkholderiales, which were introduced with wastewater (Fig. 5), due to absence of disinfection step before wastewater discharge (for details, see “ Material and methods” section) and persisted throughout the 32-h incubation, did not appear to play a significant role in functional enrichment. Nevertheless, detection of Legionella highlights the potential introduction of opportunistic pathogens into coastal systems via untreated wastewater, and that this finding warrants further research to assess persistence, ecological roles, and potential health risks.

In treatments with unfiltered wastewater, uniquely enriched proteins were mainly of Campylobacteriales (*Arcobacter*), Alteromonadales (*Marinobacter*), Oceanospirillales (*Oleiphilus*, *Hahella*), and Pseudomonadales (*Pseudomonas*) origin. They were associated with motility and signal transduction mechanisms (particularly proteins related to chemotaxis), suggesting that these functional enrichments may be related to the properties of the introduced taxa or reflect the different types of substrates and environmental signals to which they were exposed to in the unfiltered-wastewater treatment. The introduction of wastewater into coastal ecosystems likely creates chemical gradients, such as increased concentrations of attractants (e.g., nutrients) or decreasing levels of repellents (e.g., toxins) to which bacteria respond by utilizing chemotaxis [72], enabling adaptive responses to the dynamic and variable conditions introduced by wastewater. Interestingly, the dominating Alteromonas, *Glaciecola*, exhibited an increase in proteins related to drug resistance and pathogenicity (Fig. 8). Combining these findings with the previously characterized metabolic versatility of *Glaciecola* [13, 64, 81] suggests that this genus can thrive under nutrient-rich conditions, such as after wastewater discharge, while having the potential to respond to pollutants and antimicrobial compounds present in wastewater. While our data directly demonstrate the enrichment of proteins linked to resistance traits, the broader ecological implications remain inferential. We hypothesize that such traits could provide *Glaciecola* with a competitive advantage over allochthonous taxa introduced via wastewater, for example, by enhancing protection against environmental stressors or facilitating resource competition. These potential antagonistic capabilities, although not directly confirmed here, could position *Glaciecola* as an important contributor to the resilience of native microbial communities in the face of external disturbances. Other key members of wastewater degrading microbial consortia, such as Rhodobacteriales and Flavobacteriales, are also dominant groups in coastal marine environments such as the northern Adriatic [17, 61]. They utilize carbohydrates and synthesize proteins needed to meet their nutrient requirements, such as alkaline phosphatase (Figs. 3, and 7) to provide sufficient phosphorus from organophosphorus compounds in this phosphorus-limited ecosystem. Fresh wastewater-derived inorganic phosphorus input resulted in a decrease of alkaline phosphatase activity (Fig. 3), which suggests that the introduced inorganic phosphorus reduced the need for enzymatic cleavage of phosphate from organic compounds by alkaline phosphatase. The induction and repression of alkaline phosphatase synthesis by PO_4_^3−^ is a particularly well-documented example of enzyme regulation [22], relevant for phosphorus-limited ecosystems [46, 51]. Together, these results suggest that wastewater discharge can have important implications for phosphorus cycling, altering the biogeochemical state of phosphorus-limited coastal ecosystems.

A notable change in the microbiome composition in wastewater-treated microcosms was the decrease in the presence and metabolic activity of *Synechococcus*, which contributed distinct metabolic pathways in the control, including proteins related to photosynthesis (Figs. 4, 6, Supplementary File 2). This taxonomic group has previously been shown to effectively degrade antibiotics [31] and has been proposed to be suitable for wastewater bioremediation [30, 39]. The absence of *Synechococcus* in our wastewater-treated microcosms may be due to other bacteria (introduced or enriched by the wastewater addition) that could have outcompeted *Synechococcus* for available resources or occupied a similar ecological niche, or due to phages that caused a collapse in the *Synechococcus* population. However, the specific mechanisms behind this competitive exclusion and/or viral attack remain unclear, and further investigation are needed to understand the interactions among biological entities and environmental factors that might have suppressed growth of *Synechococcus* in these treatments. The impact of wastewater discharge on the abundance and metabolism of *Synechococcus* is particularly important, as this group dominates cyanobacterial communities in many coastal regions. Consequently, wastewater discharge could influence primary production, and hence, carbon cycling—one of the key services provided by marine ecosystems.

## Conclusions

We demonstrated that wastewater discharge significantly affects seawater nutrient dynamics, particularly through the introduction of dissolved organic carbon, nitrogen-compounds, and inorganic phosphorus. This nutrient influx stimulates microbial metabolic activity in the oligotrophic anthropogenically impacted coastal ecosystem. The microbiota rapidly respired wastewater-discharge, which highlights the capacity of coastal microbiomes to buffer against anthropogenic perturbations. Despite this resilience, wastewater-derived nutrients altered metabolic functions and niche utilization within the autochthonous microbial community, impacting phosphorus cycling, and potentially affecting carbon cycling*.* Although allochthonous bacteria introduced with wastewater did not significantly influence core metabolic functions or competed for niches with native taxa, they may have contributed overlooked functions beyond the current resolution of proteomic analyses. Our study focused on bacterioplankton and excluded sediments, which are known to host abundant and active microbial communities. While this represents a limitation, methodological challenges made sediment inclusion unfeasible here. Our findings highlight the critical role of microbial functional redundancy in mitigating anthropogenic perturbations, with important implications for coastal ecosystem management and wastewater impact assessments. The extent to which our findings can be generalized is constrained, however, by the high variability of wastewater treatment plant operations, effluent characteristics, and coastal marine settings. Differences in treatment efficiencies, effluent composition, trophic status, microbial community baselines, and prevailing biogeochemical conditions may all modulate microbial responses to wastewater inputs. Our results therefore provide a case-specific insight into short-term dynamics, and we caution against direct extrapolation across systems. Broader generalizations will require comparative studies across diverse wastewater sources and receiving environments.

## Material and methods

### Experimental set-up

Seawater (SW) samples were collected in the center of the Gulf of Trieste (Northern Adriatic Sea) at the location of the oceanographic buoy Vida (00BF, 45° 32′ 55.50″ N, 13° 33′ 2.52″ E) at 5 m depth on April 14, 2021. Water samples were collected with 5L Niskin samplers, stored in 1 M HCl, Milli-Q pre-washed Nalgene bottles, and immediately transported to the laboratory protected from solar radiation. In the laboratory, seawater was immediately pre-filtered through 2-µm polycarbonate filters to remove grazers (SW).

Treated wastewater was collected on April 14, 2021, at 8:10 AM at the Piran WWTP from the final holding basin where the wastewater is collected after all treatment steps, just prior to its release into the sea via underwater discharge. The WWTP Piran processes municipal and stormwater wastewater from Piran, Portorož, Lucija, Seča, Fiesa, Strunjan, and Malija, serving a combined total of 33,000 population equivalents. The system also experiences significant seawater infiltration, primarily due to leaks in the sewage infrastructure, particularly in the old town center of Piran. The plant utilizes sequencing batch reactor (SBR) technology for efficient biological treatment. Disinfection steps, such as UV treatment, are not implemented, as the outflow is discharged outside designated bathing areas. Consequently, microbiological monitoring is also not mandatory under current regulations.

After being transferred to the laboratory, collected wastewater was filtered through 5-µm polycarbonate filters to remove larger aggregates (WW-0), hereafter referred to as “unfiltered.” One part of wastewater was additionally filtered through 0.2-µm filters to remove bacterial cells (WW-F), hereafter referred to as “filtered.” Wastewater filtration (0.2 µm) was included as a mechanistic control to isolate the effects of nutrients and dissolved pollutants from the introduction of effluent-borne cells; 0.2 µm filtration removes microbial cells while preserving dissolved constituents. While 0.2 µm filtration does not remove extracellular nucleic acids, their influence on community profiles is expected to be minimal given their short persistence in seawater, their limited abundance relative to intact cells, and their low likelihood of being retained on the filters. The pH and salinity were measured to ensure that we maintained the same conditions in all experimental flasks.

For the experimental set-up, nine 5-L borosilicate glass flasks (pre-washed with 1 M HCl and Milli-Q) were used as illustrated in Fig. 1. Treatment A contained 4500 mL of seawater (SW) with the addition of 500 mL of unfiltered wastewater (WW-0) as the source of nutrients and allochthonous bacteria. Treatment B contained 4500 mL of seawater (SW) with the addition of 500 mL of 0.2 µm filtered wastewater (WW-F) as the source of nutrients. Control bottles (C) contained 5000 mL of seawater (SW). All treatments (A, B, and C) were prepared in biological triplicates. A 10% wastewater dilution was selected to mimic real-world conditions near submarine discharge of WWTP, where wastewater is rapidly diluted upon mixing with seawater. This dilution factor was determined based on a preliminary study conducted in this area [49], which showed that the initial spread of wastewater from submarine diffusers occurs at the same order of magnitude, consistent with the dilution factor proposed by the United Nations Environment Program [79].

Samples were incubated at 11.5 °C (temperature measured in situ at the time of SW sampling) in the dark. Subsampling from each bottle was performed before setting up the experiments (SW, WW-0, WW-F), immediately after the setting up the experiments (T0), and after 4 h (T1), 8 h (T2), 17 h (T3), 24 h (T4), and 32 h (TF) of incubation. Prior to each subsampling, glass flasks were gently mixed. Bacterial abundance and bacterial carbon production were determined instantly after each subsampling (see details below). At each subsampling, we collected samples for (1) microbiological analyses: bacterial abundance, bacterial carbon production (BCP), bacterial extracellular enzymes, and viral abundance; and (2) chemical analyses: dissolved organic carbon (DOC), total dissolved nitrogen (TDN), and inorganic nutrients (NH_4_^+^, NO_2_^−^, NO_3_^−^, PO_4_^3−^). In addition, subsamples of the coastal seawater (SW) and unfiltered wastewater sample (WW-0) were collected before starting the experiment, and subsamples from each experimental flask at the late exponential phase (TF) for bacterial metagenome analyses. Additionally, subsamples were taken from seawater (SW) and each experimental flask at the late exponential phase (TF) for metaproteomics analyses. Metagenomes and metaproteomes were obtained on the cellular fraction (> 0.2 µm).

### Chemical analyses

#### Dissolved organic carbon and nitrogen

Samples for dissolved organic carbon (DOC) were filtered onto pre-combusted Whatman GF/F filters (~ 0.8 µm pore size) into pre-combusted glass vials and stored at − 30 °C until analysis. DOC and TDN (total dissolved nitrogen) analyses were performed by a high-temperature catalytic method using a Shimadzu TOC-L analyzer equipped with a total nitrogen unit [37, 73]. The instrument was calibrated using potassium phthalate whereas quality control was ensured by the analysis of a certified surface seawater reference material (Consensus Reference Material, University of Miami, FL). The method is characterized by a precision of < 3% expressed as %RSD.

#### Dissolved inorganic nutrients

Dissolved inorganic nitrogen (NH_4_^+^, NO_2_^−^, NO_3_^−^) and dissolved inorganic phosphorus (PO_4_^3−^) concentrations were determined spectrophotometrically by segmented flow analysis (QuAAtro, Seal Analytical) following standard methods [38]. The validation and accuracy of the results were checked with reference material (KANSO CO., LTD.) before and after sample analyses. The quality control is performed annually by participating in an inter-calibration program (QUASIMEME Laboratory Performance Study).

### Viruses, bacterial abundance, and bacterial carbon production

The abundance of bacteria and virus-like particles (VLPs) was estimated by flow cytometry. Samples (1 mL) were fixed with 0.5% (final concentration) glutaraldehyde solution (Grade I for EM analyses, Sigma Aldrich). Fixed samples were kept at 4 °C for approximately 15 min and then stored at − 80 °C until analysis. Prior to enumeration, samples were thawed at room temperature and diluted 1:10 for bacterial abundance and 1:50 for VLPs with 0.2 μm-filtered Tris–EDTA buffer 1X (Sigma Aldrich). Samples for VLPs were stained with SYBR Green I nucleic acid dye (0.5 × 10^−4^ dilution of the commercial stock; Life Technologies) and incubated in the dark at 80 °C for 15 min. Samples for bacterial abundance were stained with SYBR Green I nucleic acid dye (1 × 10^−4^ dilution of the commercial stock; Life Technologies) and incubated in the dark at room temperature for 10 min according to Marie et al. [52]. Data were acquired with a FACSCanto II (Becton Dickinson) flow cytometer equipped with an air-cooled laser at 488 nm and standard filter set-up and processed with the FACSDiva software (Becton Dickinson). VLP abundance was obtained by correcting the total count for noise, with 0.2 μm filtered Tris–EDTA buffer 1X (Sigma Aldrich) as blank [12]. The flow rate was calibrated daily using distilled water and weighing it before and after the run (at least 5 replicates). Bacterial and viral abundances were then calculated using the acquired cell counts and the respective flow rates.

Bacterial carbon production (BCP) was determined using radiolabeled leucine (^3^H-Leu) incorporation into newly synthesized proteins [45, 71]. The triplicates of each sample were incubated with ^3^H-Leu (Perkin Elmer) at 20 nM final concentration at 15 °C in the dark for 1 h. Thereafter, TCA (trichloroacetic acid) (5% final concentration) was added to stop cell growth. The samples were centrifuged and cleaned according to the protocol of Smith and Azam [71]. Scintillation liquid (Ultima Gold, PerkinElmer) was added and the radioactivity was measured using a liquid scintillation counter (Canberra Packard TriCarb Liquid Scintillation Analyzer, model 2500 TR). The bacterial production was calculated as described by Simon and Azam [70] using a theoretical leucine-to-carbon conversion factor 3.1 kg C mol^−1^ Leu.

Bacterial growth rate was calculated from the log of bacterial abundance during exponential growth (between 8 and 32 h of incubation). Assuming that bacterial metabolism was only fueled by DOC, the decrease in DOC was used as a proxy for the heterotrophic bacterial carbon demand (BCD). The BCD represents the sum of carbon consumed for the synthesis of new bacterial biomass (bacterial production, BP) and the amount of organic carbon respired (bacterial respiration, BR). We estimated bacterial production also from the increase of bacterial biomass (BB) over time (calculated from the differences in bacterial abundance assuming C-content of 19.8 fg C cell^−1^ [47]. From the BCD and bacterial production, we estimated the bacterial growth efficiency (BGE = BP/BCD). Sample C2 was excluded from these calculations since there was no observed decrease in DOC concentration.

### Extracellular enzymatic activity

Extracellular enzymatic activities were assayed using fluorogenic substrate analogues [41] derived from 7-amino-4-methyl-coumarin (AMC) and 4-methyl-umbelliferone (MUF). Leucine aminopeptidase activity was assayed as the hydrolysis rate of leucine-AMC. β-glucosidase, olease, chitinase, and alkaline phosphatase activities were assayed using MUF-β-D-glucoside, MUF-oleate, MUF-*N*-acetyl-β-d-glucosaminide, and MUF-phosphate, respectively (Sigma Aldrich®).

Hydrolysis was measured by incubating 0.3 mL sub-samples with 200 μM MUF-β-D-glucoside, MUF-*N*-acetyl-β-D-glucosaminide, leucine-AMC, 100 μM MUF-oleate, and 50 μM MUF-phosphate in the dark at the experimental temperature (saturating concentrations, [16]) for 1 h. Fluorescence increases due to MUF and AMC hydrolyzed from the model substrate were measured using a Spark 20 M (Tecan) spectrofluorometer (MUF = 365-nm excitation and 455-nm emission,AMC = 380-nm excitation and 440-nm emission). All samples were run in five replicates. Triplicate calibration curves were performed using 0.2 μm filtered seawater or wastewater and 5 μM MUF and AMC standard solutions.

### Bacterial metagenomes

A large volume of seawater (~ 1 L) was filtered onto 0.2-µm polyethersulphone filters (PALL Life Sciences), and stored at − 80 °C until further processing. Genomic DNA was extracted from the filters using phenol–chloroform extraction protocol according to Angel [4], with slight modifications for the use of filters as described in Bayer et al. [11], and then sequenced with an Illumina NovaSeq (2*150 bp) platform at Mycrosynth AG (Balgach, Switzerland).

The initial quality check of raw forward and reverse reads was done using the FastQC tool [3]. Reads were quality filtered with FastP v0.20.1 [21] with qualified_quality_phred 20 (the quality value that a base is qualified), unqualified_percent_limit 20 (20% of bases are allowed to be unqualified), and required length of 50 bp. Co-assembly of contigs was processed using Spades tool (v. 3.15.2) [9] with default parameters. Contigs were prepared to import in Anvi’o by simplifying names with anvi-script-reformat-fasta command, where only contigs with a minimal length of 1000 bp were kept. Further metagenome analyses were performed using Anvi’o v7 [27]. A contig database was generated with anvi-gen-contigs-database command, which includes gene calling with Prodigal [42]. Gene calls were analyzed using the Hidden Markov model (hmm). Contig database statistics were obtained with the anvi-display-contigs-stats program. Nucleotide and amino acid sequences were extracted with anvi-get-sequences-for-gene-calls. Genes were annotated with functions from NCBI’s Clusters of Orthologous Groups with anvi-run-ncbi-cogs command. anvi-run-kegg-kofams was used to annotate functions and metabolic pathways in a contigs database using KEGG database. The taxonomic composition of gene calls was annotated with Kaiju v1.7.3, which relies on NCBI taxonomy, and imported into contig database with anvi-import-taxonomy-for-genes. Single-copy gene taxonomy was run with anvi-run-scg-taxonomy, which relies on The Genome Taxonomy Database GTDB [62]. A single-copy gene, Ribosomal S2, was selected for this analysis due to its consistently high coverage across all samples. Reads of each sample were separately mapped to the co-assembly using Bowtie2 and output was converted in.bam files using samtools. An anvi’o profile database was generated from individual bam files and contigs database using anvi-profile command. Profiles were merged into single profile database with anvi-merge.

Binning of the co-assembled metagenome into metagenome-assembled genomes (MAGs) was performed by the “anvi-cluster-contigs” program using two different binning algorithms (metabat2 and concoct) [2, 44]. Binning results of both algorithms were aggregated with DAS Tool [69]. Metagenome-assembled genomes (MAGs) quality was evaluated with two complementary approaches. First, completeness and redundancy were checked with CheckM [63]. Second, within the anvi’o framework, we applied anvi-estimate-genome-completeness, which reports genome completeness and redundancy—a metric equivalent to contamination—using its curated single-copy core gene sets. MAGs were retained for downstream analyses if they satisfied at least one of the following quality criteria: (i) ≥ 80% completeness and ≤ 5% redundancy according to anvi’o, or (ii) ≥ 80% completeness and ≤ 5% contamination according to CheckM. Gene coverages and “detection” in the tested samples were extracted using anvi-export-gene-coverage-and-detection. Taxonomic classification of MAGs was done with Gtdb-Tk tool [19], which classifies genomes based on Genome Taxonomy Database (GTDB) [62]. Visualization of MAGs was done using anvi-interactive.

Selected *Glaciecola* genomes (Supplementary Table S4) obtained from GenBank were annotated as described above and the pangenome was reconstructed using the Anvio program “anvi-pangenome” [25] with MCL hierarchical clustering of the genes [80]. The average nucleotide identity was calculated using PyANI [65].

Since different databases were used for gene-calls taxonomic classification and compared to single-copy genes and MAG taxonomic classification (NCBI and GTDB, respectively), we noticed differences in taxonomic classification results on different levels of our analyses. However, this was considered in the interpretation and discussion of the results.

### Metaproteomics

#### Protein extraction

We extracted proteins from bacterial biomass collected on 0.2-µm pore-size hydrophilic PVDF Durapore® (Millipore) filters. Seawater samples (2 L) were collected before the start of the experiment and from all experimental treatments (A1 = 0.9 L, B1 = 1.5 L, C2 & C3 = 1.1 L, other samples = 1 L) at the peak of prokaryotic abundance (32 h). All samples were kept at − 80 °C until processing. Filters were placed into tubes and ground into small pieces with a sterile metal spatula after the tubes were submerged in liquid nitrogen. Protein extraction is presented in detail in Supplementary Text S2.

#### LC–MS measurements and data analysis

Purified tryptic peptides were dissolved in 0.1% formic acid (FA) and measured using a nano-reversed-phase high-performance liquid chromatography (RP-HPLC) system (Thermo Scientific Dionex Ultimate 3000), coupled with a benchtop Quadrupole Orbitrap mass spectrometer (Q-Exactive Plus, Thermo Scientific). Peptides were separated on a PepMap RSLC C18 column at 55 °C with a flow rate of 300 nL/min. The 2-h segmented LC–MS gradient ranged from 5 to 80% buffer B (79.9% acetonitrile, 0.1% FA). Mass spectra were acquired in positive ion mode using a top-15 data-dependent acquisition method. Full MS scans were performed at a resolution of 70,000 (*m/z* 200), followed by MS/MS scans at a resolution of 17,500. High-energy collisional dissociation (HCD) fragmentation was applied with a normalized collision energy (NCE) of 30%. Dynamic exclusion was set to 60 s.

Proteomic data were analyzed using the Proteome Discoverer v2.2.0.388 (ThermoFisher Scientific, Rockford, IL, USA) at the Life Science Computer Cluster (LiSC) of the University of Vienna (as previously described in [11], and [76]).

Protein identification was performed by searching the MS data using SEQUEST-HT against the bacterial protein-coding genes of the co-assembled metagenome. Search parameters were as follows: enzyme—trypsin, fragment mass tolerance—0.8 Da, max. missed cleavages—2, fixed modifications—carbamidomethyl (Cys), optional modifications—oxidation (Met). Percolator parameters were as follows: max. delta Cn: 0.6, max. rank: 0, validation based on *q*-value, false discovery rate of 1% (calculated by automatic decoy searches). Protein abundance was calculated based on the total area under the chromatographic peaks of unique and razor peptides.

#### Protein enrichment analyses

For protein enrichment analyses, standard differential expression analysis was applied using *DEseq2* package [48]. Shrinking of effect size was done on log2fold change values with *lfcShrink* function using “*apeglm*” shrinkage estimator [83]. An enrichment algorithm was applied to test the differences between each pair of treatments (A vs C, B vs C, and A vs B). Proteins with corrected *p*-value < 0.01 were considered significant and included in the results of enrichment analysis. Proteins were aggregated based on corresponding COG functional categories, where the mean log2fold change was calculated.

To determine the relative contribution of each taxon to each COG category, every peptide was assigned a lowest common ancestor (LCA) of each top BLAST hit for the metagenome proteins containing the peptide (Anvi’o). Nonbacterial PSMs were removed from further analysis.

#### Statistical analyses and data visualization

All the statistics and visualization were performed in R v4.0.5 (R Cole Team 2022) using RStudio v2022.07.2. The metagenomic and metaproteomic data sets were combined and managed using “phyloseq” v1.34 [56]. Dissimilarities between samples and statistical tests were carried out using “vegan” v2.5–7 [60]. Plotting was carried out using “ggplot2” v3.3.3 [35] and “networkD3” v0.4 [1].

## Supplementary Information

Supplementary Material 1.

Supplementary Material 2.

Supplementary Material 3.

Supplementary Material 4.

## Data Availability

Raw sequencing data for this study have been deposited in the European Nucleotide Archive (ENA) at EMBL-EBI under accession number PRJEB85785 (https:/www.ebi.ac.uk/ena/browser/view/PRJEB85785). The mass spectrometry proteomics data have been deposited to the ProteomeXchange Consortium (Deutsch et al., 2023) via the PRIDE (Perez-Riverol et al., 2025) partner repository with the dataset identifier PXD061573. All scripts used for data processing are available in the Github repository https:/github.com/Orel-N/WW_MicrocosmExp.
