# Supplementary material for: Down the drain: exploring wastewater’s role in coastal microbiome transformations
Source: Microbiome. 2025 Dec 24;14:46. doi: 10.1186/s40168-025-02298-1 (PMC12849601; doi:10.1186/s40168-025-02298-1)
Supplement: Supplementary file 2 — Supplementary Material 1. [file 40168_2025_2298_MOESM1_ESM.docx]

***
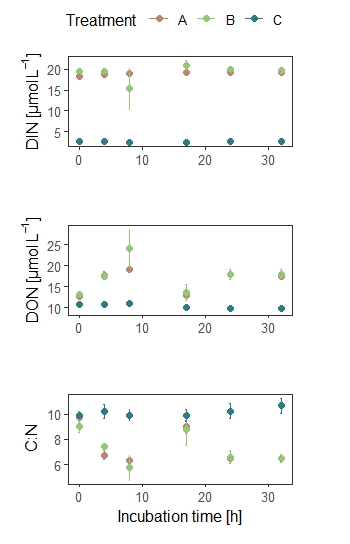
***

***Supplementary figure 1:*** Dynamics of dissolved inorganic nitrogen (DIN), dissolved organic nitrogen (DON) and C:N ratio during 32 h of incubation in treatments A (seawater with unfiltered wastewater addition), B (seawater with filtered wastewater addition) and control C (seawater). Each value represents the mean of three replicates ± SE.


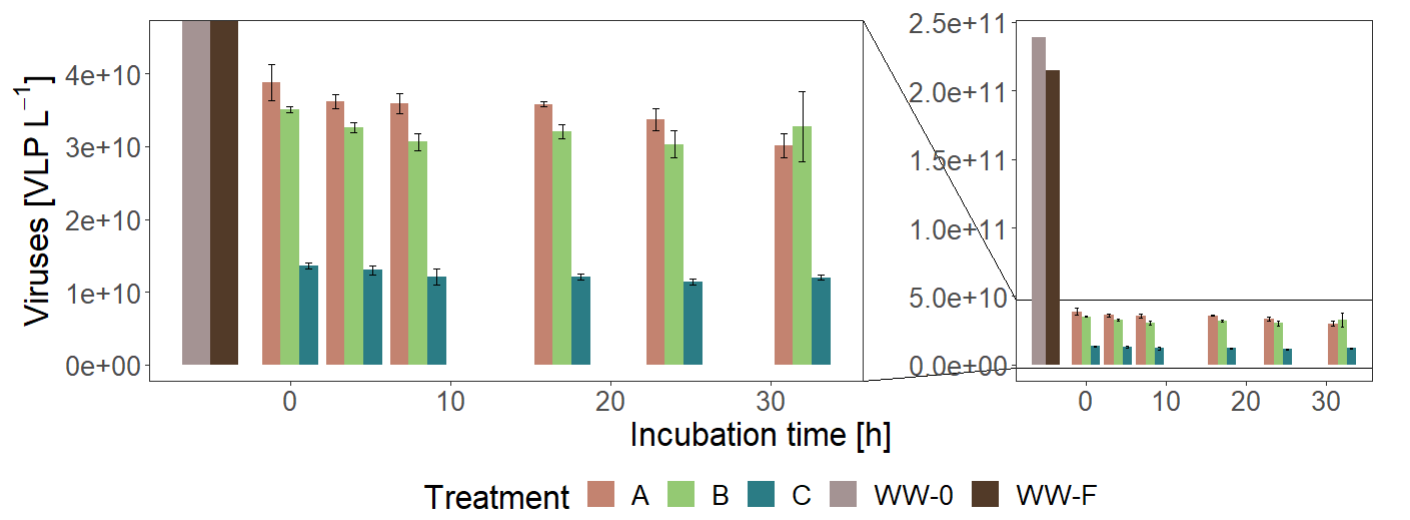


***Supplementary figure 2:*** Dynamic of virus like particles (VLP) prior to incubation in WW-0 (unfiltered wastewater) and WW-F (filtered wastewater) and during 32 h incubation in treatments A (seawater with unfiltered wastewater addition), B (seawater with filtered wastewater addition) and control C (seawater). Each value represents the mean of three replicates ± SE.

***
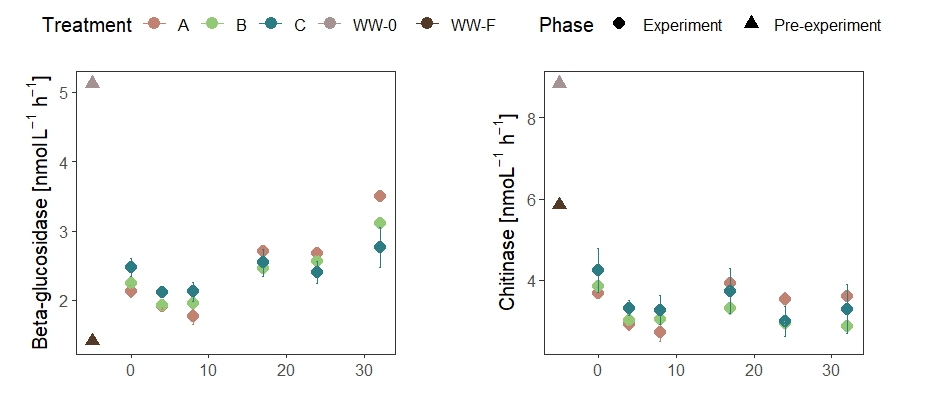
***

***Supplementary figure 3:*** Dynamics of β-glucosidase and chitinase determined using fluorogenic substrate analogues (c) prior to incubation in WW-0 (unfiltered wastewater) and WW-F (filtered wastewater) and during 32 h incubation in treatments A (seawater with unfiltered wastewater addition), B (seawater with filtered wastewater addition) and C (seawater). Each value represents the mean of three replicates ± SE.

**
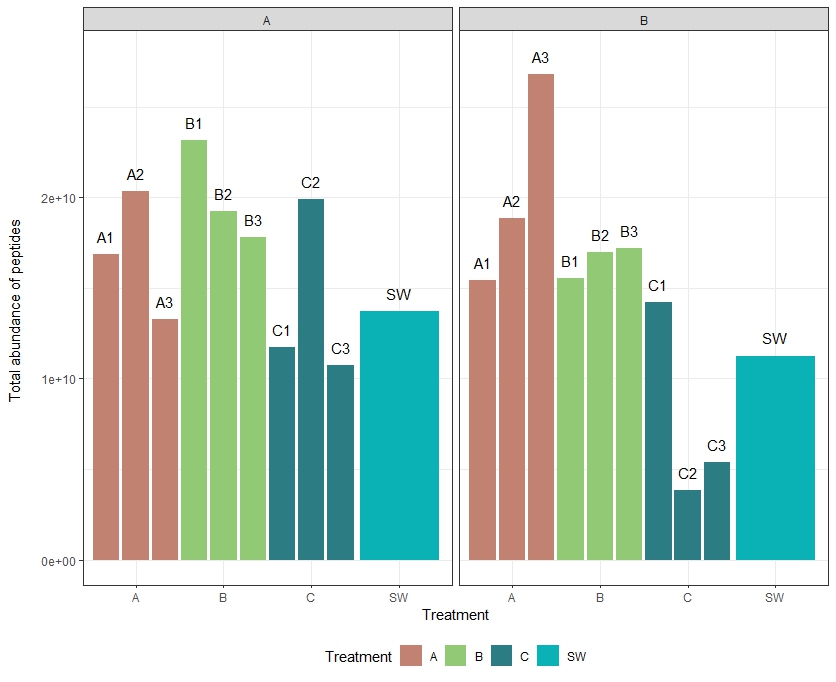
**

***Supplementary Figure 4:*** Total abundance of peptides detected across different runs. Panels show protein counts, comparing Run A and Run B across treatments (A, B, C, and SW).

**
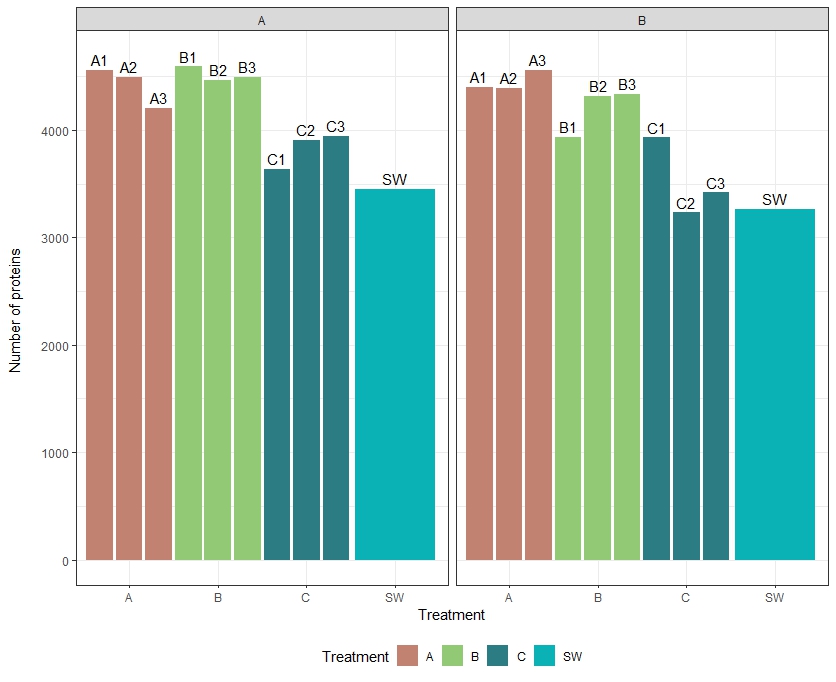
**

***Supplementary Figure 5:*** Total number of proteins detected across different runs. Panels show protein counts, comparing Run A and Run B across treatments (A, B, C, and SW).


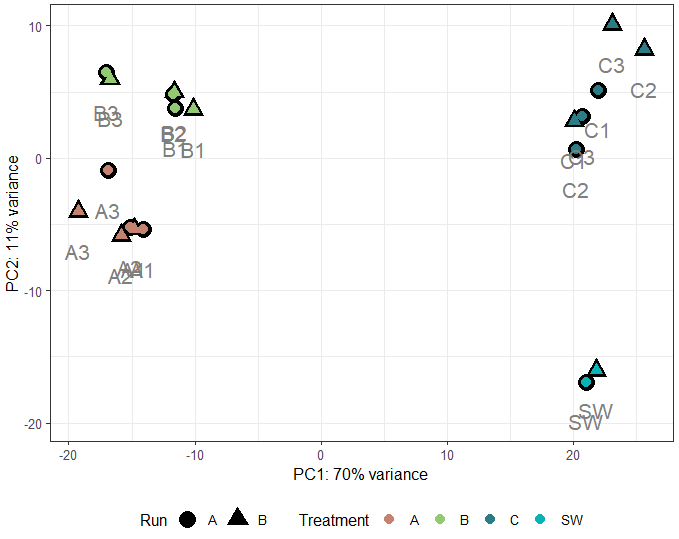


| **Adonis2** | **Df** | **SumOfSqs** | **R2** | **F** | **Pr(>F)** |
| --- | --- | --- | --- | --- | --- |
| **Treatment** | **3** | **16592** | **0.63021** | **9.0919** | **0.001** |
| **Run** | **1** | **611** | **0.02322** | **1.0048** | **0.343** |

***Supplementary Figure 6:*** Dissimilarity of the cellular protein composition in different treatments and initial seawater sample measured in Run A and Run B. Principal component analysis (PCA) was performed on variance-stabilized protein composition.


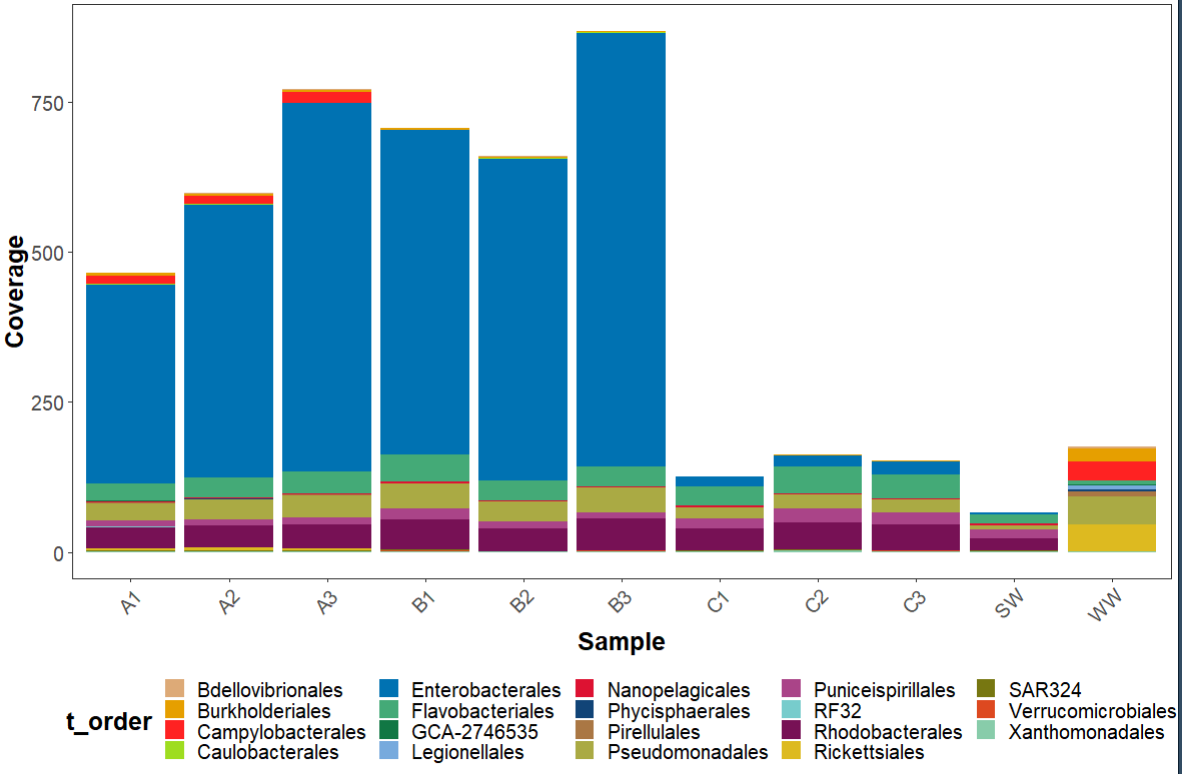


***Supplementary Figure 7:*** Read coverage of metagenome-assembled genomes (MAGs) across different samples (A1, A2, A3, B1, B2, C1, C2, SW, WW). Stacked bars represent the relative contribution of different taxonomic orders (t_order), as indicated in the legend.


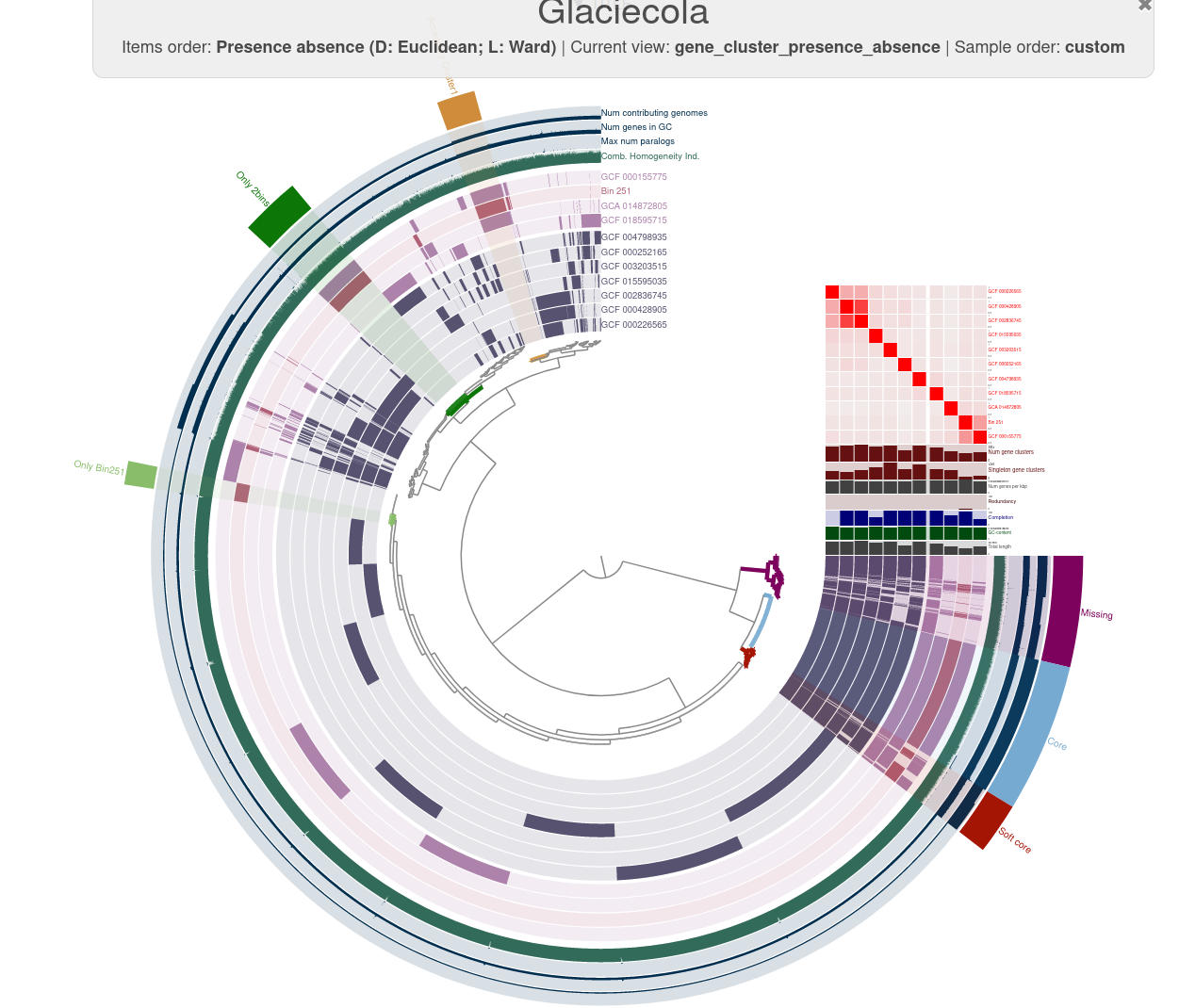
***Supplementary Figure 8:*** Pangenome analysis of *Glaciecola*. The heatmap represents average nucleotide identity (95-100%), ordered according to phylogenetic tree. ‘Bin_251’ represents MAG (metagenome assembled genome) from this study, other genomes are closest related reference genomes (based on GTDB-Tk).


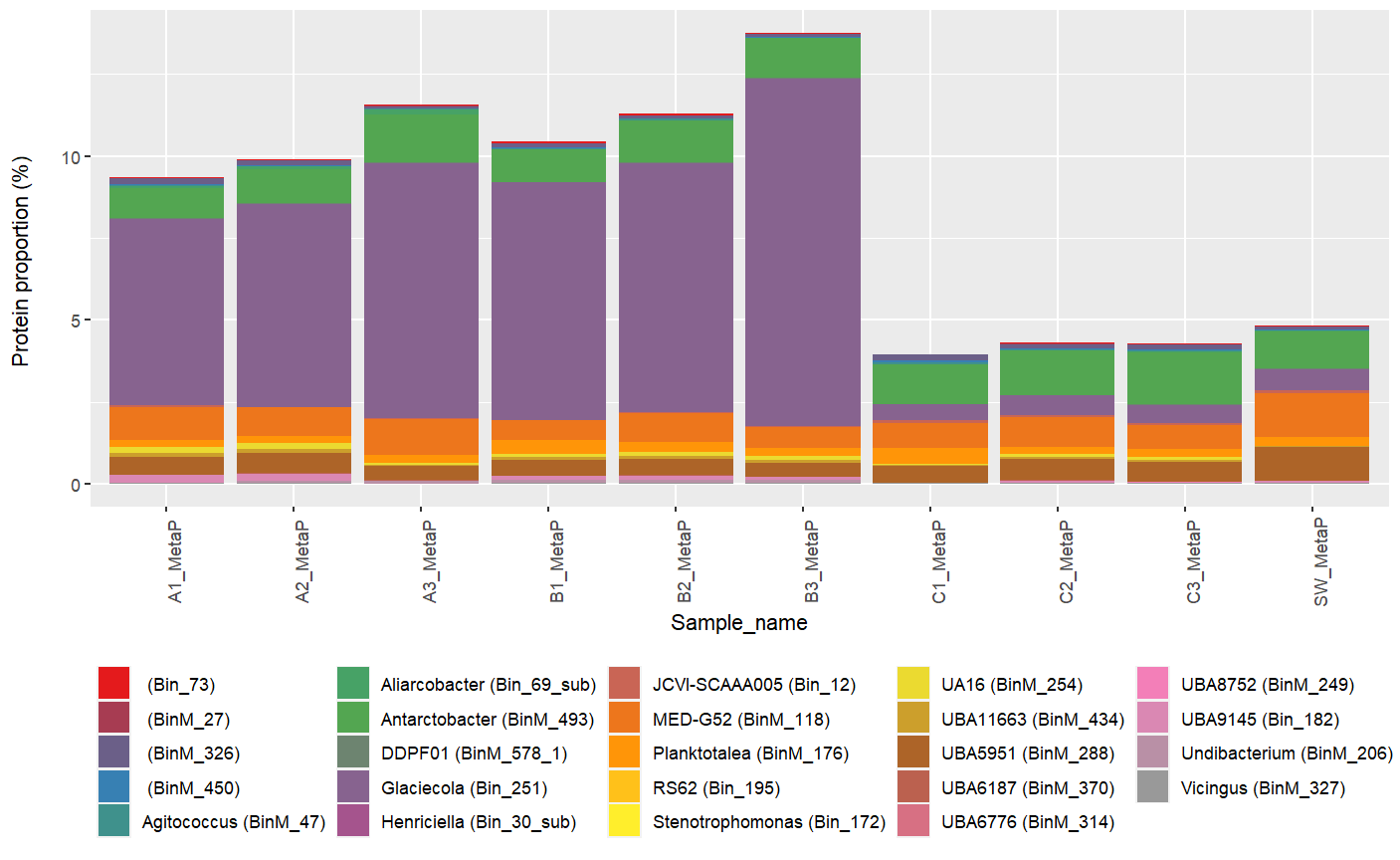
***Supplementary Figure 9:*** Proportion of proteins assigned to metagenome-assembled genome (MAG) bins across samples (A1, A2, A3, B1, B2, B3, C1, C2, C3, and SW). Stacked bars represent the relative contribution of proteins associated with individual bins as identified in the legend.
