# Supplementary material for: Down the drain: exploring wastewater’s role in coastal microbiome transformations
Source: Microbiome. 2025 Dec 24;14:46. doi: 10.1186/s40168-025-02298-1 (PMC12849601; doi:10.1186/s40168-025-02298-1)
Supplement: Supplementary file 3 — Supplementary Material 2. [file 40168_2025_2298_MOESM2_ESM.docx]

**Supplementary Text S1: Technical details of metagenomic and metaproteomic datasets and analyses**

Shotgun sequencing of 11 samples generated 269.2 Gb of raw data (average 24.5 Gb per sample). Quality filtering resulted in 22.6 Gb of filtered sequencing data per sample (**Supplementary table 1**). We performed co-assembly (722369 contigs, total length 2.1 Gb) (**Supplementary table 2**), followed by binning. Co-assembly was used to produce a protein sequence database to which all tandem mass peptide spectra were correlated.

We identified 2.9 × 10^10^ ± 7.3 × 10^9^ peptides per sample in metaproteomes, that could be matched to 7217 ± 707 predicted coding sequences of metagenomes (**Supplementary figure 4, Supplementary figure 5**). Peptides were measured in two runs (run A, run B), which resulted in a comparable number of detected proteins (**Supplementary Figure 5**). Adonis test showed that there was no significant difference between runs (PERMANOVA test, F=1.0, R2=0.02, p=0.343, **Supplementary Figure 6**). For the following analyses only Run A was selected. The composition of proteins was significantly different between treatments (PERMANOVA test, F=9.1, R2=0.63, p=0.001, **Supplementary figure 6**). The number of proteins was similar among biological triplicates, and slightly lower in the control and seawater inoculum than in the wastewater-treated microcosms (**Supplementary figure 5**).

**Supplementary Text S2: Detailed Methodology: Description of sample preparation and analysis of proteomic data**

For analysis of the metaproteome filter pieces were resuspended in lysis buffer (100mM Tris-HCl pH 7.4, 1% SDS, 150mM NaCl, 1mM DTT, 10mM EDTA) and cells were lysed with five freeze-and-thaw cycles. After centrifugation (10,000x *g* at 4°C for 25 min) the supernatant was transferred into a tube and proteins were co-precipitated with 0.015% deoxycholate and 7% trichloroacetic acid (TCA) on ice for 1h and washed once with ice-cold acetone. Dried protein pellets were resuspended in 50 mM TEAB buffer (Millipore Sigma, Burlington, MA, USA) and quantified using Pierce 660 nm Protein Assay Reagent (ThermoFisher Scientific, Rockford, IL, USA). The concentration of proteins ranged from 1.32 μg μL^-1^ to 2.3 μg μL^-1^.

Cysteines were reduced and alkylated with 10 mM DTT and 55 mM iodoacetamide (IAA), respectively, as described by (Shevchenko et al., 2006). Proteins were re-precipitated using 9 volumes of 96% EtOH at -20°C overnight prior to overnight trypsin (Roche) digestion (1:100, w/w) at 37°C according to Valledor and Weckwerth (2014). Trypsin digestion was terminated by adding trifluoroacetic acid (TFA) to the samples (1% final concentration). Samples were desalted using Pierce C18 Tips (Thermo Scientific) according to the manufacturer’s protocol.

**References:**

Bayer, B., Pelikan, C., Bittner, M. J., Reinthaler, T., Könneke, M., Herndl, G. J., et al. (2019). Proteomic Response of Three Marine Ammonia-Oxidizing Archaea to Hydrogen Peroxide and Their Metabolic Interactions with a Heterotrophic Alphaproteobacterium. *mSystems* 4. doi: 10.1128/mSystems.00181-19

Shevchenko, A., Tomas, H., Havlis, J., Olsen, J. V., and Mann, M. (2006). In-gel digestion for mass spectrometric characterization of proteins and proteomes. *Nat Protoc* 1, 2856–2860. doi: 10.1038/nprot.2006.468

Valledor, L., and Weckwerth, W. (2014). An improved detergent-compatible gel-fractionation LC-LTQ-Orbitrap-MS workflow for plant and microbial proteomics. *Methods Mol Biol* 1072, 347–358. doi: 10.1007/978-1-62703-631-3_25
